# Evaluating the Clinical Validity of Hypertrophic Cardiomyopathy Genes

**DOI:** 10.1161/CIRCGEN.119.002460

**Published:** 2019-02-19

**Authors:** Jodie Ingles, Jennifer Goldstein, Courtney Thaxton, Colleen Caleshu, Edward W. Corty, Stephanie B. Crowley, Kristen Dougherty, Steven M. Harrison, Jennifer McGlaughon, Laura V. Milko, Ana Morales, Bryce A. Seifert, Natasha Strande, Kate Thomson, J. Peter van Tintelen, Kathleen Wallace, Roddy Walsh, Quinn Wells, Nicola Whiffin, Leora Witkowski, Christopher Semsarian, James S. Ware, Ray E. Hershberger, Birgit Funke

**Affiliations:** 1 Agnes Ginges Centre for Molecular Cardiology at Centenary Institute and Faculty of Medicine and Health, The University of Sydney, University of Sydney, Australia (J.I., C.S.).; 2 Department of Cardiology, Royal Prince Alfred Hospital, Sydney, Australia (J.I., C.S.).; 3 Department of Genetics, UNC Chapel Hill, NC (J.G., C.T., E.W.C., S.B.C., J.M., L.V.M., B.A.S., N.S., K.W.).; 4 Stanford Center for Inherited Cardiovascular Disease, Stanford University, CA (C.C.).; 5 Eastern Virginia Medical School, Norfolk, VA (K.D.).; 6 Laboratory for Molecular Medicine, Partners Healthcare, Harvard Medical School, Cambridge, MA (S.M.H.).; 7 Division of Human Genetics, Davis Heart and Lung Research Institute (A.M., R.E.H.); 8 Division of Cardiovascular Medicine, The Ohio State University, Columbus (R.E.H.).; 9 Oxford Medical Genetics Laboratory, United Kingdom (K.T.).; 10 Department of Clinical Genetics, Amsterdam University Medical Centers, University of Amsterdam, Cardiovascular Sciences, The Netherlands (J.P.v.T.).; 11 National Heart and Lung Institute & MRC London Institute of Medical Sciences, Imperial College London, United Kingdom (R.W., N.W., J.S.W.).; 12 Cardiovascular Research Centre at Royal Brompton & Harefield Hospitals NHS Trust, London, United Kingdom (R.W., N.W., J.S.W.).; 13 Department of Medicine, Vanderbilt University Medical Center, Nashville, TN (Q.W.).; 14 Department of Pathology, Harvard Medical School/Massachusetts General Hospital, Boston (L.W., B.F.).

**Keywords:** genetic testing, heart failure, syndrome, uncertainty

## Abstract

**Background:**

Genetic testing for families with hypertrophic cardiomyopathy (HCM) provides a significant opportunity to improve care. Recent trends to increase gene panel sizes often mean variants in genes with questionable association are reported to patients. Classification of HCM genes and variants is critical, as misclassification can lead to genetic misdiagnosis. We show the validity of previously reported HCM genes using an established method for evaluating gene-disease associations.

**Methods:**

A systematic approach was used to assess the validity of reported gene-disease associations, including associations with isolated HCM and syndromes including left ventricular hypertrophy. Genes were categorized as having definitive, strong, moderate, limited, or no evidence of disease causation. We also reviewed current variant classifications for HCM in ClinVar, a publicly available variant resource.

**Results:**

Fifty-seven genes were selected for curation based on their frequent inclusion in HCM testing and prior association reports. Of 33 HCM genes, only 8 (24%) were categorized as definitive (*MYBPC3*, *MYH7*, *TNNT2*, *TNNI3*, *TPM1*, *ACTC1*, *MYL2*, and *MYL3*); 3 had moderate evidence (*CSRP3, TNNC1*, and *JPH2*; 33%); and 22 (66%) had limited (n=16) or no evidence (n=6). There were 12 of 24 syndromic genes definitively associated with isolated left ventricular hypertrophy. Of 4191 HCM variants in ClinVar, 31% were in genes with limited or no evidence of disease association.

**Conclusions:**

The majority of genes previously reported as causative of HCM and commonly included in diagnostic tests have limited or no evidence of disease association. Systematically curated HCM genes are essential to guide appropriate reporting of variants and ensure the best possible outcomes for HCM families.

## Abstract 2

Supplemental Digital Content is available in the text.

Hypertrophic cardiomyopathy (HCM) is an inherited cardiomyopathy, characterized by left ventricular hypertrophy (LVH) in the absence of loading conditions such as hypertension.^1,2^ HCM affects ≈1 in 500 in the general population^3,4^ with clinical features in patients ranging from asymptomatic to heart failure and sudden cardiac death. Since the first chromosomal location was mapped in 1989,^5^ variants in numerous genes have been reported to cause HCM. Clinical diagnostic genetic testing for HCM has become increasingly part of mainstream clinical management of patients^6–8^ with a key role in cascade testing of family members. Genotype positive relatives can be targeted for ongoing cardiac screening while genotype negative relatives can be released from life-long surveillance and worry.^8^ Although there is potential for HCM genetic testing to add significant value to family management, nonjudicious use has potential for harm including variant misclassification and genetic misdiagnosis.^9^

The implementation of next-generation sequencing has led to a rapid expansion in the number of genes included in a typical diagnostic gene panel. Gene selection, in addition to increased stringency and expert classification of variants,^8–11^ is a crucial but often overlooked first step. Identification of variants in genes with limited gene-disease association has the potential to add uncertainty and misinterpretation of variants as causative.^12–14^ Here, we examine the evidence supporting 57 genes included on diagnostic HCM gene panels using the National Institutes of Health-funded Clinical Genome Resource (ClinGen) framework for evaluating gene-disease clinical validity.^15,16^ Although other recent studies have investigated specific aspects of HCM gene association such as gene burden, we bring together all available evidence in a systematic way. We evaluate the quantity and quality of clinical genetic and experimental data using a scoring matrix and give a final overall summary classification. In addition, we use the public repository ClinVar,^17^ to cross-reference HCM variant classifications and to examine the impact of including insufficiently supported genes in clinical testing.

## Methods

All data and materials have been made publicly available on the ClinGen website https://www.clinicalgenome.org/working-groups/clinical-domain/cardiovascular-clinical-domain-working-group/hypertrophic-cardiomyopathy-gene-ep/ and can be accessed at URL in the Data Supplement. No institutional review board approval was required. The full Methods are available in the Data Supplement.

## Results

### Selection of the Gene List

Of 328 gene panels identified in the National Center for Biotechnology Information Genetic Testing Registry, 24 were included (Table I in the Data Supplement; 20 panels identified as HCM panels and 4 cardiomyopathy panels). The mean (±SD) number of genes per panel was 33±25, including 8±0.3 sarcomere genes, 4±2 storage cardiomyopathy, and RASopathy genes (*LAMP2*, *PRKAG2*, *TTR*, *GLA*, *PTPN11*, *RIT1*, and *RAF1*) and 22±23 other genes (range, 0–75). There were 162 unique genes represented across all panels (Figure II in the Data Supplement; Table II in the Data Supplement). Only 23 (14%) genes were present on >50% of the panels. All panels included key sarcomere genes (*MYBPC3*, *MYH7*, *TNNT2*, *TNNI3*, *TPM1*, *ACTC1*, *MYL2*, and *MYL3*), except one that did not include *MYH7*. The final curation list included 57 genes (Tables III and IV in the Data Supplement); 33 were curated for HCM and 24 for syndromes or conditions including LVH (Figure III in the Data Supplement; Table V in the Data Supplement). Twenty-six of the 57 genes had prior reported association with HCM in Online Mendelian Inheritance in Man (http://www.omim/org; Tables VI and VII in the Data Supplement).

### Classification of HCM Genes

**Figure 1.**
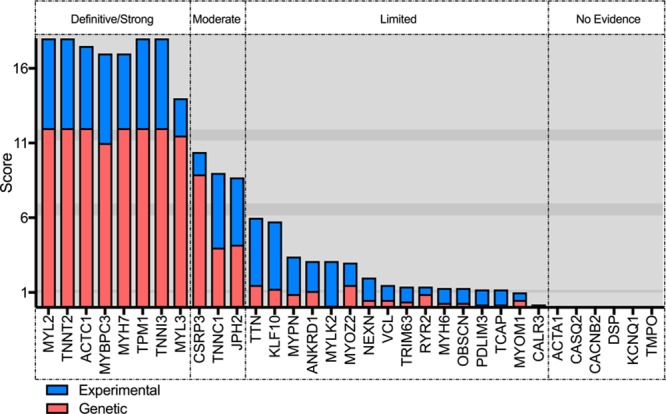
**Hypertrophic cardiomyopathy gene-disease classifications showing genetic, experimental, and overall scores and classification.**

Of the 33 genes classified for HCM, 8 (24.2%) were classified as definitive, 3 (9.1%) as moderate, 16 (48.5%) as limited, and 6 (18.2%) as no evidence (Figure 1). Those classified as definitive included well-known disease genes that have been included in diagnostic gene panels for over a decade (*MYBPC3*, *MYH7*, *TNNT2*, *TNNI3*, *TPM1*, *ACTC1*, *MYL3*, and *MYL2*). All definitive genes primarily reached this classification because of genetic evidence reflecting numerous reports from the literature of causative variants in cases with HCM. Furthermore, all genes had some variants with strong segregation data and an aggregate variant excess in cases compared with controls (Table 1).^12^ Moderate level gene classifications included *TNNC1, JPH2*, and *CSRP3*, with evidence typically including either segregation evidence or reported *de novo* variants and some experimental evidence (Table 2). The majority (n=22; 66.7%) of genes had limited or no evidence of HCM association. Limited evidence genes typically included evidence from candidate gene studies, with observation of rare variants in cases, but without statistical evidence of an excess of rare variation in cases compared with background variation in controls, or segregation data, and minimal experimental evidence mostly from expression data. In some cases, limited evidence was available from animal models (*KLF10*, *MYOZ2*, and *MYPN*).

**Table 1.**
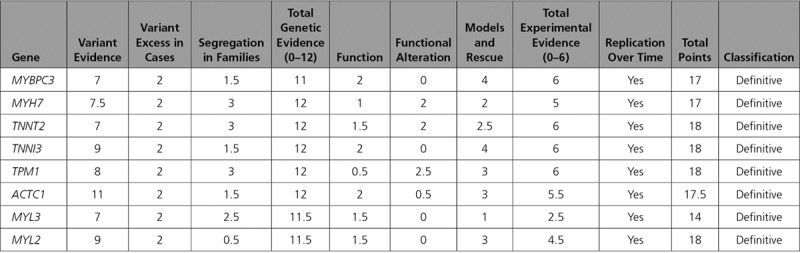
Definitive Classifications for Hypertrophic Cardiomyopathy Gene Associations

**Table 2.**
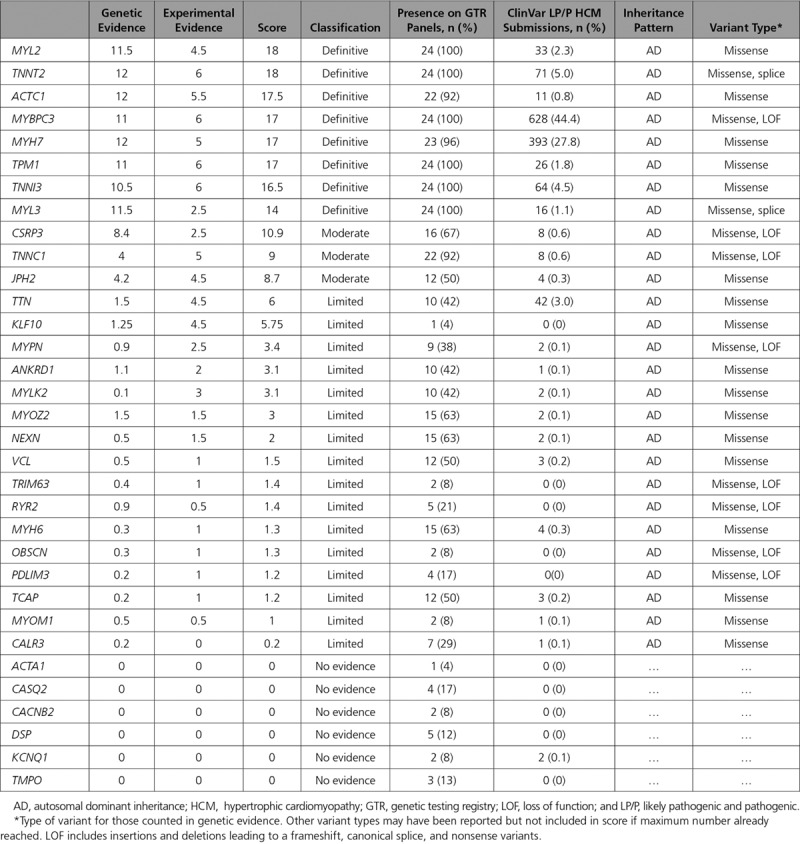
Genetic, Experimental, and Overall Classifications for Genes Curated for HCM

### Impact of Including HCM Candidate Genes in Diagnostic Gene Panels

**Figure 2.**
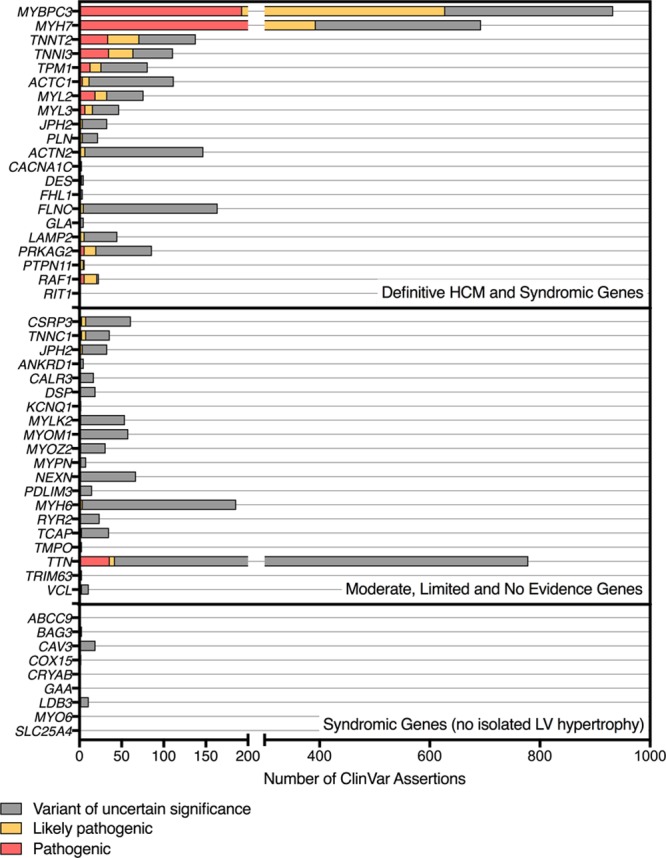
**Number of ClinVar assertions for hypertrophic cardiomyopathy (HCM) phenotype, grouped by gene classifications.** LV indicates left ventricle.

The American College of Medical Genetics and Genomics and the Association for Molecular Pathology variant classification framework requires substantial evidence for a gene-disease association to assign a pathogenic or likely pathogenic classification to variants identified in any specific gene.^10^ The inclusion of insufficiently supported genes in diagnostic testing practice increases the likelihood of inconclusive results being provided to clinicians. Moreover, this effect can be pronounced when these genes have a high rate of population variation. To investigate this, we analyzed ClinVar variant entries for the HCM genes included in our curation effort. This resulted in 4191 assertions for variants in 50 genes (Table VIII in the Data Supplement). Of all assertions, 831 (20%) were classified as pathogenic, 584 (14%) were likely pathogenic, and 2776 (66%) were variants of uncertain significance (VUS) (Figure 2; Table IX in the Data Supplement). There were 65 (5%) variants in genes with limited or no evidence classified as likely pathogenic or pathogenic, with most (n=38) being truncating or splice variants in *TTN*, which are known to occur at high frequency (≈1%) in the general population. In total, 1252 VUS assertions were in HCM genes adjudicated as limited or no evidence and accounted for 30% of all assertions in ClinVar meeting analysis criteria for this study.

### Classification of Syndromic Genes

Twenty-four genes were curated for syndromes involving LVH, with the specific syndrome the gene was curated for shown in Table 3. Two genes (*ACTN2* and *PLN*) best fit an intrinsic (primary) cardiomyopathy phenotype given there were no extracardiac features reported. *PLN* reached a definitive classification, with the phenotype spectrum including HCM, arrhythmogenic right ventricular cardiomyopathy, and dilated cardiomyopathy.^18^ *ACTN2* reached a moderate classification, with the reported phenotypes including HCM, left ventricular noncompaction, atrial arrhythmias, and idiopathic ventricular fibrillation.^19,20^ Dilated cardiomyopathy cases were carefully reviewed to exclude end-stage HCM.

**Table 3.**
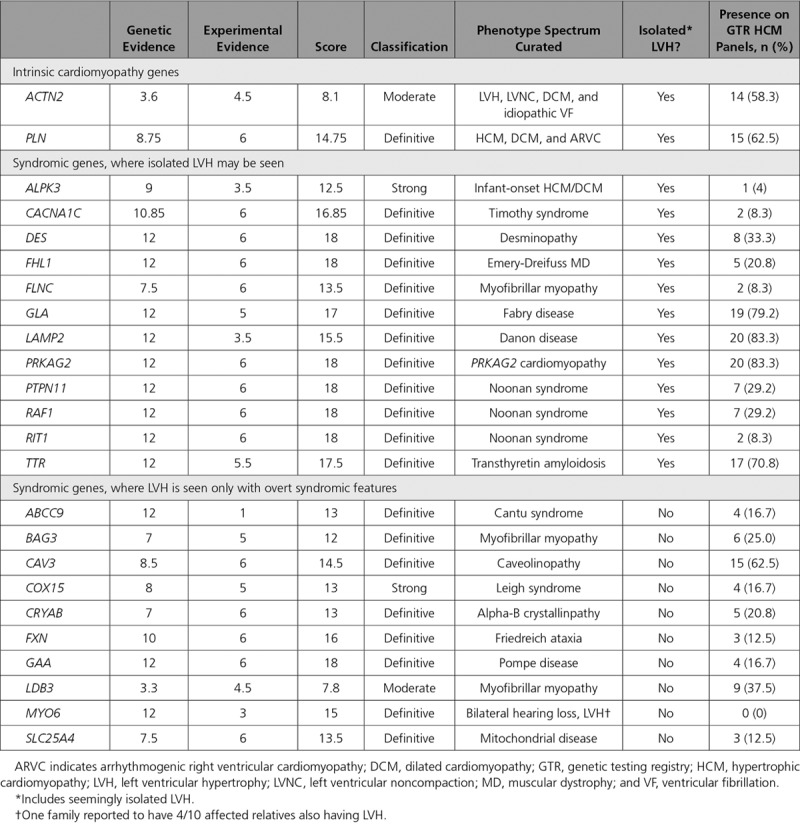
Genetic, Experimental, and Overall Classifications for Genes Curated for Syndromes Including LVH

Twelve genes were curated for syndromes that may present with isolated, or seemingly isolated, LVH. Of those, 11 were classified as having a definitive association with their respective syndromes. *CACNA1C* is associated with Timothy syndrome. Infants may present with severe biventricular hypertrophy, and 2 variants at a single amino acid position (p.Arg518Cys and p.Arg518His) have been reported to occur in families with LVH, prolonged QT interval, and sudden cardiac death.^21^ Variants in *DES* cause a desminopathy, often occur *de novo* and are associated with a range of features including progressive skeletal muscle weakness, cardiomyopathy, including LVH in some cases, and cardiac conduction disease.^22^ *FHL1* has been shown to cause Emery-Dreifuss muscular dystrophy, which can include LVH, even in the absence of significant muscle weakness.^23^ *FLNC* causes a myofibrillar myopathy, though families with isolated LVH are known.^24,25^ Four genes, all associated with metabolic storage phenotypes that can mimic HCM (*GLA*: Fabry disease, *LAMP2*: Danon disease, *PRKAG2*: PRKAG2 cardiomyopathy, and *TTR*: transthyretin amyloidosis), were classified as definitive. *PTPN11*, *RAF1*, and *RIT1* are associated with Noonan syndrome and were classified as definitive.^26^ Autosomal recessive loss-of-function variants in *ALPK3* cause a severe infant-onset cardiomyopathy and were classified as strong.^27^

Ten genes were curated for syndromes that may include LVH only in combination with overt extracardiac phenotypic features, therefore making variants in these genes unlikely to be reasonably mistaken for typical HCM (Table 3).

## Discussion

We report a systematic classification of genes commonly included in HCM genetic testing or previously reported as HCM genes in the public domain, using the ClinGen gene curation clinical validity framework.^15^ Two-thirds of the curated genes had limited or no evidence of HCM association. We observed little consistency among currently offered diagnostic gene panels, likely influenced by the lack of widely accepted systematic curation efforts and absence of clear guidelines about the design of clinically valid gene panels. Reporting a VUS may cause confusion, especially to clinicians less sanguine in understanding their marginal utility for clinical care. As genetic testing enters mainstream medical practice, discontinuity between evidence in support of specific gene-disease associations and the interpretation and use of variant data can be minimized with robust systematic gene classification efforts.

Our findings have direct clinical implications for the genetic evaluation and care of HCM families. We show that in ClinVar, a large publicly accessible database, nearly 30% of assertions made for HCM in our curated gene list were VUS in genes with limited or no evidence of HCM association. This illustrates VUS inflation because of reporting of variants in these genes. Genetic test results are probabilistic and some degree of uncertainty is inherent, though efforts should be made to minimize this where possible in the clinical setting, focusing on results that are meaningful to the clinician, patient, and family. Given the uncertainty we face in understanding the significance of rare variation in numerous genes, the reporting of VUS in genes with tenuous HCM association is at best unnecessary and time-consuming, and at worst has potential to inflict harm to families who may be over-investigated or inappropriately treated,^9,28^ especially in the absence of specialized disease-specific expertise. As predictive testing becomes mainstream, it will be increasingly important to consider the evidence supporting a disease association to avoid misclassification of uncertain variants that can lead to unnecessary medical action.

Clearly defined boundaries are needed for when candidate genes should be used for clinical testing. The current plethora of genes purported to be associated with HCM is the result of dedicated efforts to better elucidate its genetic architecture. A decade ago, the promise of identifying new genes to explain gene-negative HCM cases was the driver of numerous candidate gene studies. At that time a missense variant in a conserved region, absent in a small set of control alleles, and with some evidence of segregation, suggested a new HCM gene association. Such an approach is now widely recognized as insufficient^10,29^; however, published disease associations have led to inclusion of such genes on clinical test panels.

Diagnostic gene panels should include genes considered to have definitive or strong evidence of disease association to minimize the risk of inconclusive findings. Moderately associated genes should be considered more carefully, though variants may be considered causative if there is very clear supportive evidence of a functional or damaging effect for the variant. Inclusion of genes with limited or no evidence for a disease association can be useful when the availability of large pedigrees allows clarification of uncertain variants identified in the proband, or when multiple family member exome or genome testing is used for clinically challenging cases. However, these applications currently straddle the boundary between diagnostic and research testing and their benefit needs to be carefully weighed against potential negative impact to the patient.

Given isolated LVH due to a syndrome may be confused with a diagnosis of HCM, inclusion of genes with moderate level and above for syndromes leading to isolated LVH is warranted. Where causative variants in syndrome genes are identified, concordance with the extracardiac phenotype features is important. Defining a precise cause of LVH, by identifying a genetic cause implicating an HCM mimic, has direct clinical advantages in many cases. For example, more targeted therapies such as enzyme replacement therapy in Fabry disease, or in guiding prognosis in young males with Danon disease. Appropriate recognition and implementation of such information by genetic testing groups and clinical genetics professionals are needed, and we suggest this process as a basis for laboratories to aid in designing gene panels that match their intended use. Increasingly, panels are configured to evaluate several disorders with clinical and genetic overlap, however, this approach is likely to result in greater identification of VUS.

Study limitations include genes potentially not curated in this iteration. However, our systematic approach to selecting genes ensured that we included most with a published association with HCM and those commonly included on panels. The ClinGen gene curation framework assumes a Mendelian mode of inheritance; more complex models, should they be relevant, exceeded this analysis paradigm. Gene classifications provided were based on available evidence at the time of curation; ongoing and updated reassessments of gene-disease associations are essential. Importantly, our framework relies on variant curation, and whereas the difficulties in determining variant pathogenicity have been well documented,^12,13,30,31^ we relied on variant curation best practices as foundational to this effort. The materials and framework to perform gene curations are publicly available. Summaries for the gene-disease evidence assessments are available online (https://search.clinicalgenome.org/kb/gene-validity) and in Document II in the Data Supplement.

## Conclusions

HCM genetic testing has entered mainstream medical care. HCM genetic testing has important benefits for asymptomatic at-risk relatives and in the future may play a role in prognostic and therapeutic stratification of the proband. Our findings highlight that most reported HCM genes are spurious, including many genes routinely included in current diagnostic panels, with profound implications for the risk of genetic misdiagnosis in HCM families. Robust international gene curation efforts, as described here, bring together many types of evidence and are essential to yield the greatest value from HCM genetic testing.

## Acknowledgments

With many thanks to Brandi Kattman, MS, CGC, Staff Scientist / Genetic Counselor, National Institutes of Health Genetic Testing Registry for sharing data on genes present on hypertrophic cardiomyopathy next-generation sequencing panels. We thank various groups within ClinGen including the RASopathy Expert Panel and Hearing Loss Working Group for input on genes of interest; the Lumping and Splitting Working Group for guidance on decisions about disease entities to curate; Heidi Rehm, PhD for advice, and the Gene Curation Working Group (co-chairs Jonathan Berg, MD, PhD, and Christa Martin, PhD, FACMG) for helpful discussions and guidance on the gene-disease clinical validity framework.

## Sources of Funding

Dr Ingles is the recipient of a National Heart Foundation of Australia Future Leader Fellowship (number 100833). Dr van Tintelen acknowledges the support from the Netherlands Cardiovascular Research Initiative, an initiative with support of the Dutch Heart Foundation (CVON2014-40 DOSIS). K. Thomson is funded by a National Institute for Health Research (NIHR) and Health Education England Healthcare Science Doctoral Research Fellowship (NIHR-HCS-D13-04-006). Dr Semsarian is the recipient of a National Health and Medical Research Council Practitioner Fellowship (number 1059156). Dr Hershberger and A. Morales were supported by ClinGen subcontracts (National Human Genome Research Institute [NHGRI]; NHGRI HG007437 and NHGRI 1U41HG009650). We are very grateful to the NHGRI for funding this work (grant number U01HG007437-04, U41HG009650, and U41HG006834.). This study was supported by the Wellcome Trust (107469/Z/15/Z); Medical Research Council (UK); NIHR Royal Brompton Biomedical Research Unit; and NIHR Imperial Biomedical Research Centre.

## Disclosures

None.

## Supplementary Material
