# Supplementary material for: Evaluating the Clinical Validity of Hypertrophic Cardiomyopathy Genes
Source: Circ Genom Precis Med. 2019 Feb 19;12(2):e002460. doi: 10.1161/CIRCGEN.119.002460 (PMC6410971; doi:10.1161/CIRCGEN.119.002460)
Supplement: Supplementary file 1 [file hcg-12-e002460-s001.pdf]

SUPPLEMENTAL MATERIAL

METHODS:

Personnel

Curations were performed and reviewed by a group of 23 individuals from 10 institutions and 4 countries (Australia, Netherlands, UK, and USA), either with significant clinical, molecular diagnostic and/or research experience in HCM, or biocurators with expertise in the ClinGen gene-disease clinical validity framework, who worked as 'curation pairs' for gene curation. The group was a subgroup of the ClinGen Cardiovascular Clinical Domain Working Group (https://www.clinicalgenome.org/working-groups/clinical-domain/cardiovascular-clinical-domain-working-group/).

Selection of gene curation list

We considered two criteria for selecting genes, those previously reported to have an association with HCM or isolated LVH and those frequently included on molecular diagnostic HCM gene panels.

A gene list was assembled using the following sources:

(1) Presence on >30% of "HCM" gene panels listed by National Center for Biotechnology Information (NCBI) Genetic Testing Registry (Supplementary Figure 1).

The NCBI Genetic Testing Registry was queried on March 16, 2016 using search terms "hypertrophic" or "cardiomyopathy". One panel per laboratory was selected, and where laboratories offered multiple cardiac panels, the one most targeted to HCM was included (Supplementary Tables 1 and 2).

(2) Presence on the Illumina Cardio TruSight gene panel.[1]

A comprehensive list of reported HCM genes based on reported disease association in the Human Gene Mutation Database and manual curation of literature (Supplementary Table 3).

(3) Expert knowledge by the authors.

Additional genes were included, such as recently published HCM genes, and based on experience with patient gene reports (Supplementary Figure 2).

Curated phenotypes

Here we refer to HCM as isolated LVH, in the absence of other extra-cardiac features.[2, 3] The term "LVH" is used to denote a phenotypic feature that is part of a syndromic presentation.

Given the need for our gene list to address potential HCM genocopies, we curated two categories of gene-disease pairs: (1) HCM, and (2) syndromic disease, where LVH is reported as part of a wider phenotypic spectrum. This included phenotypes with variable cardiomyopathy features and/or arrhythmia, herein termed intrinsic (primary) cardiomyopathy, or those where LVH has been reported to occur either in isolation, or where other features can be subtle or manifest later and therefore appear to present as isolated LVH. Where evidence suggested that the phenotypic spectrum may include multi-system features, preliminary curations were considered according to ClinGen's recommendations (https://www.clinicalgenome.org/working-groups/lumping-and-splitting/). For example, while *MYH7* was curated for HCM, *FLNC* was curated for myofibrillar myopathy, which in some cases can present as isolated LVH.

Gene curation process

Curation pairs performed a literature review, assessed the clinical and experimental evidence for each disease-gene association, and made a provisional classification. The full review group met twice monthly by conference call to discuss curated genes and to establish final classifications according to ClinGen's Clinical Validity framework.[4] Gene curations were performed according to the ClinGen Clinical Validity Gene Curation Process Standard Operating Procedure version 5, November 2017 (current version at: https://www.clinicalgenome.org/curation-activities/gene-disease-validity/educational-and-training-materials/standard-operating-procedures/; version used available in Supplementary Document 1).

Evaluated genetic evidence primarily included published case-level data. Only variants shown to be absent or very rare in the Exome Aggregation Consortium (ExAC) or Genome Aggregation (gnomAD) databases were counted (minor allele frequency <1:1000). Segregation studies contributed additional points, based on the reported or estimated LOD score. Gene-level case-control data were used for genes where available.[5, 6]

Experimental evidence was assessed under the categories of function, functional alteration, and models and rescue (Supplementary Material). Function assessed whether the encoded protein has a function consistent with the known mechanisms of HCM, interacts with proteins previously implicated in HCM, or is expressed in cardiac tissue. "Functional alteration" assessed whether variants found in patients with disease have been shown to alter function of the protein in cell culture models and/or patient cells. Model systems assessed whether disruption to the gene of interest in non-human animal or cell culture models recapitulated a phenotype (or endophenotype) of HCM, and rescue assessed whether cell culture models or non-human animal models with an HCM phenotype could be rescued by restoring the wild-type gene product. Functional evidence was only included for variants that remained sufficiently rare in ExAC and gnomAD.

ClinGen's point-based scoring matrix was used to arrive at a final summary score and genes were classified as no evidence of disease-gene association (score=0), limited (>0-6), moderate (7-11) and strong (12-18). Genes that reached a classification of strong for which evidence was replicated over time (>3 years since the original publication and >2 publications supporting the gene-disease association) were classified as definitive. Links to the HCM gene-disease clinical validity classifications and written summaries are provided in the Supplementary Material.

Publicly reported HCM variant classifications.

The ClinVar (http://www.ncbi.nlm.nih.gov/clinvar) database was queried April 19, 2018 for the phenotype/condition including “hypertrophic” or “HCM” for all variant assertions (pathogenic, likely pathogenic and variant of uncertain significance (VUS)), and those for the 57 genes were analyzed. Only assertions where the variant had been identified in an individual with HCM, or the variant previously reported in HCM patients, were included.

RESULTS

ClinVar Variant Assertions for the Curated Gene List

There were 6128 ClinVar variant assertions (i.e. separate entries) identified where the phenotype nominated by the submitter contained the term “hypertrophic” or “HCM”. Assertions not relating to our curated gene list, a non-HCM phenotype (e.g. hypertrophic osteoathropathy), or a variant classification other than pathogenic, likely pathogenic or VUS were removed, leaving 4191 assertions for variants in 50 genes (Supplementary Table 9). Of all assertions, 831 (19.8%) were classified as pathogenic, 584 (14.0%) were likely pathogenic and 2776 (66.2%) were VUS (Figure 3; Supplementary Table 9). There were 65 (4.6%) likely pathogenic or pathogenic assertions for variants in genes with “limited” or “no evidence” of association, including 42 assertions for variants in *TTN* (38 truncating or splice variants, and 26 from a single submitter). In total, likely pathogenic or pathogenic assertions were made for variants in 42 genes, including 9 genes (21.4%) considered ’Definitive‘ or ’Strong”, 3 (7.1%) ’Moderate‘ and 12 (28.6%) with ’Limited’ or ’No evidence‘ for an association with HCM. The remaining likely pathogenic or pathogenic assertions were for variants in 12 (28.6%) genes where LVH can be a presenting feature, and 6 (14.3%) syndrome genes. Most likely pathogenic and pathogenic variant assertions were for *MYBPC3* (n=628) and *MYH7* (n=393).

In total, 1252 VUS assertions were in genes with ’Limited‘ or ’No evidence‘ of HCM association, and accounted for 30% of all assertions in ClinVar meeting the inclusion criteria. VUS assertions were most frequently reported for *TTN* (n=737), *MYBPC3* (n=305), *MYH7* (n=300), *MYH6* (n=182) and *FLNC* (n=159). Those 17 ’Limited‘ or ’No evidence‘ HCM genes that had VUS assertions on ClinVar were further assessed to compare missense constraint scores compared to ’Definitive‘ HCM genes. Those with ’Limited‘ or ’No evidence‘ of HCM association showed greater tolerance to missense variation (ExAC missense constraint z-scores: 0.35 ± 2.3 versus 2.6 ± 2.2, p=0.037), indicating the genes showing “VUS inflation” are more tolerant to rare variation than ’Definitive‘ classified genes.

REFERENCES

1. Pua CJ, et al. Development of a Comprehensive Sequencing Assay for Inherited Cardiac Condition Genes. *J Cardiovasc Transl Res*. 2016;9:3-11.

2. Gersh BJ, et al. 2011 ACCF/AHA Guideline for the Diagnosis and Treatment of Hypertrophic Cardiomyopathy: a report of the American College of Cardiology Foundation/American Heart Association Task Force on Practice Guidelines. Developed in collaboration with the American Association for Thoracic Surgery, American Society of Echocardiography, American Society of Nuclear Cardiology, Heart Failure Society of America, Heart Rhythm Society, Society for Cardiovascular Angiography and Interventions, and Society of Thoracic Surgeons. *J Am Coll Cardiol*. 2011;58:e212-260.

3. Elliott PM, et al. 2014 ESC Guidelines on diagnosis and management of hypertrophic cardiomyopathy: the Task Force for the Diagnosis and Management of Hypertrophic Cardiomyopathy of the European Society of Cardiology (ESC). *Eur Heart J*. 2014;35:2733-2779.

4. Strande NT, et al. Evaluating the Clinical Validity of Gene-Disease Associations: An Evidence-Based Framework Developed by the Clinical Genome Resource. *Am J Hum Genet*. 2017;100:895-906.

5. Walsh R, et al. Reassessment of Mendelian gene pathogenicity using 7,855 cardiomyopathy cases and 60,706 reference samples. *Genet Med*. 2016.

6. Walsh R, et al. Defining the genetic architecture of hypertrophic cardiomyopathy: re-evaluating the role of non-sarcomeric genes. *Eur Heart J*. 2017.

Supplementary Tables and Figures:

For supplementary tables, please see included excel spreadsheets.

- S Table 1: Full list of unique genes identified on n=24 NCBI Genetic Testing Registry panels
- S Table 2: 24 GTR panels showing percentage of panels representing the gene
- S Table 3: Gene lists showing compilation of the final curation gene list
- S Table 4: Full list of curated genes showing ExAC missense and loss of function constraint scores and GTEx expression in left ventricle
- S Table 5: Full list of curated genes and matrix scores
- S Table 6: Full list of curated genes and OMIM phenotype description
- S Table 7: 26 OMIM genes reported to be associated with "HCM"
- S Table 8: All n=4191 ClinVar assertions identified using the search strategy
- S Table 9: ClinVar assertions by classification and gene
- S Material Document Contains:
    - More detailed Methods and Results
    - Supplementary Figure 1: NCBI Genetic Testing Registry (GTR) panels meeting inclusion criteria, showing types of genes included
    - Supplementary Figure 2: Development of the gene curation list
    - Supplementary Figure 3: Flowchart of the gene list and gene classifications
- S Document 1: ClinGen Gene Clinical Validity Curation Process Standard Operating Procedure version 5, November 2017
- S Document 2: HCM Gene Curation Summaries

SUPPLEMENTARY FIGURE 1

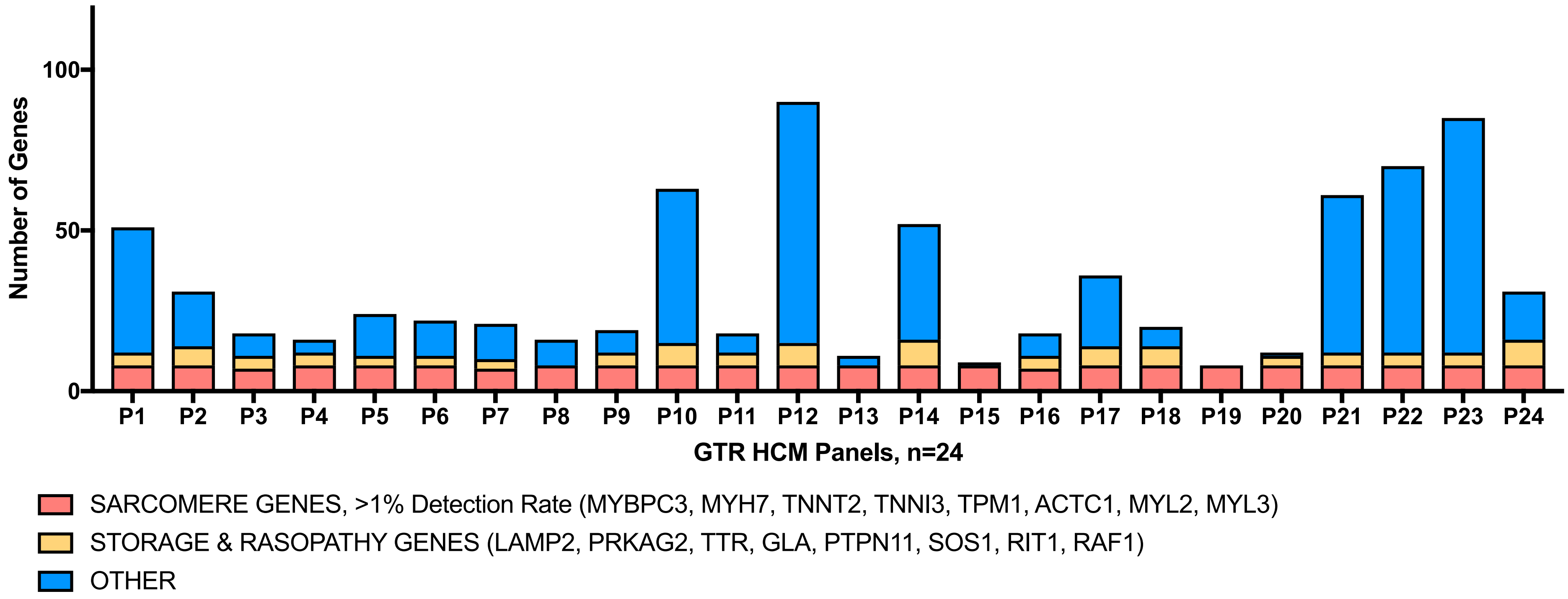

SUPPLEMENTARY FIGURE 1: NCBI Genetic Testing Registry (GTR) panels meeting inclusion criteria, showing types of genes included. Details shown in supplementary tables.

SUPPLEMENTARY FIGURE 2

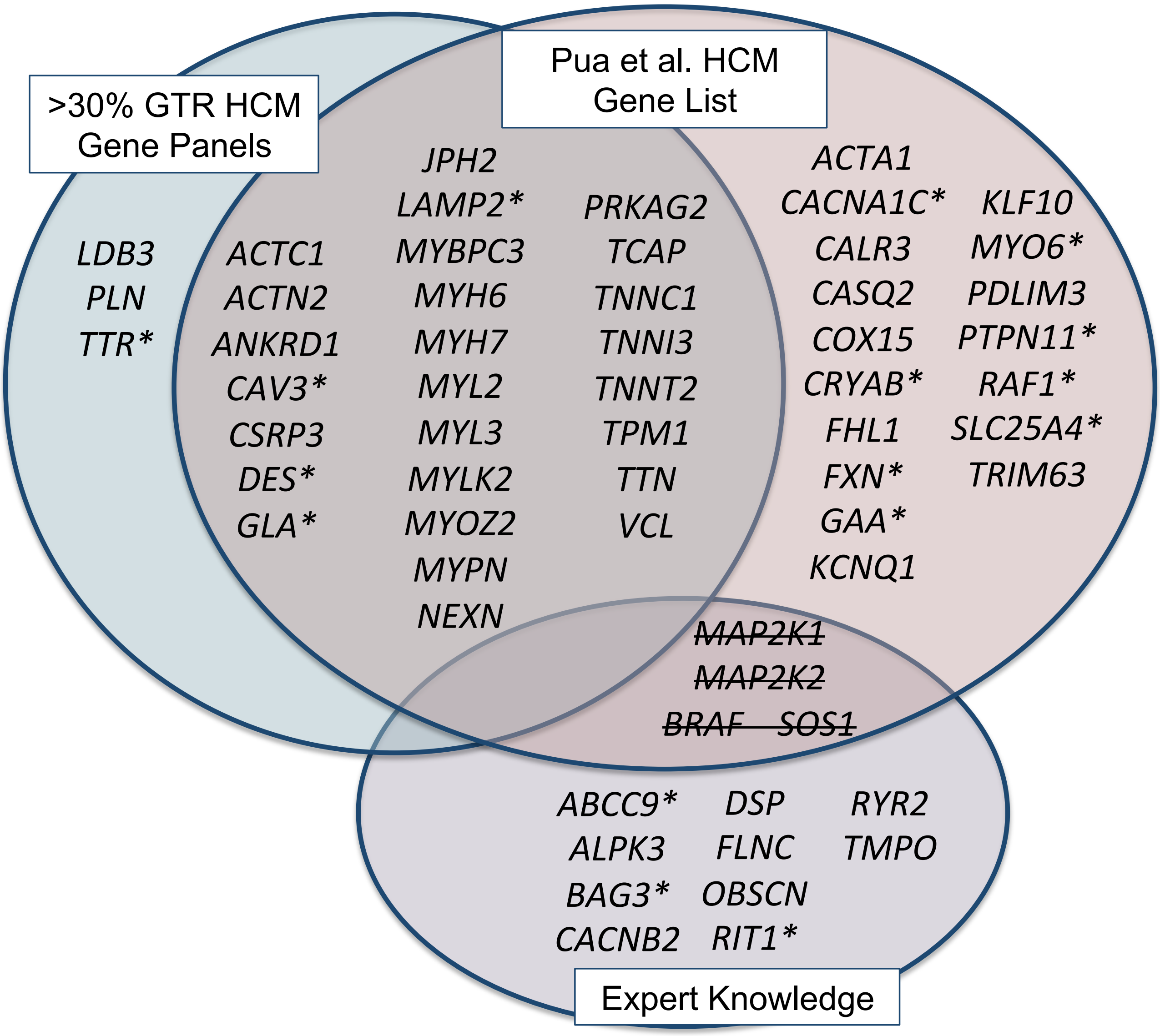

SUPPLEMENTARY FIGURE 2: Development of the gene curation list. Source of the genes are as indicated. *Genes curated by an alternate curator/ expert pair: *ABCC9, GAA, GLA*, Goldstein/Berg; *CAV3, COX15, CRYAB, FXN, LAMP2, TTR*, Thaxton/Berg; *DES*, McGlaughon/Berg; *BAG3*, Strande/Berg; *CACNA1C,* Thaxton/Channelopathy Expert Panel; *MYO6*, Hearing Loss Expert Panel; *PTPN11, RIT1, RAF1*, RASopathy Expert Panel. Four genes were removed from the curation.

SUPPLEMENTARY FIGURE 3

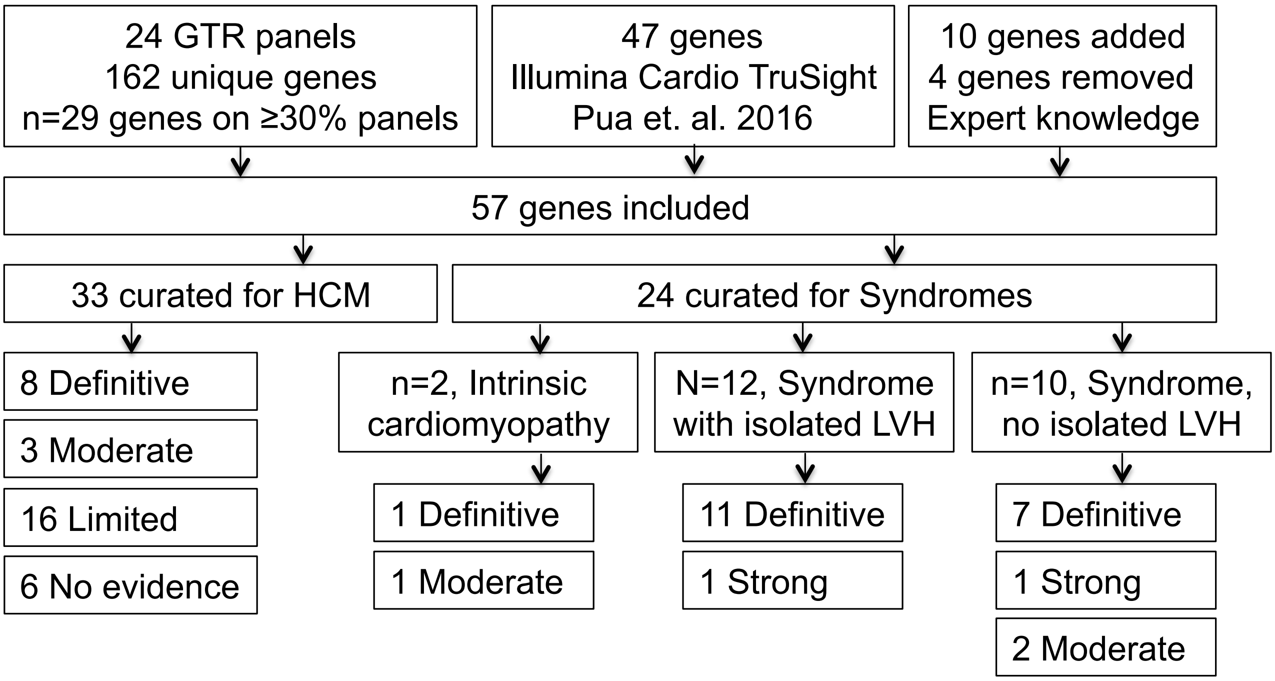

SUPPLEMENTARY FIGURE 3: Flowchart of the gene list and gene classifications

Abbreviations: GTR. Genetic testing registry; LVH, left ventricular hypertrophy

SUPPLEMENTARY FIGURE 3

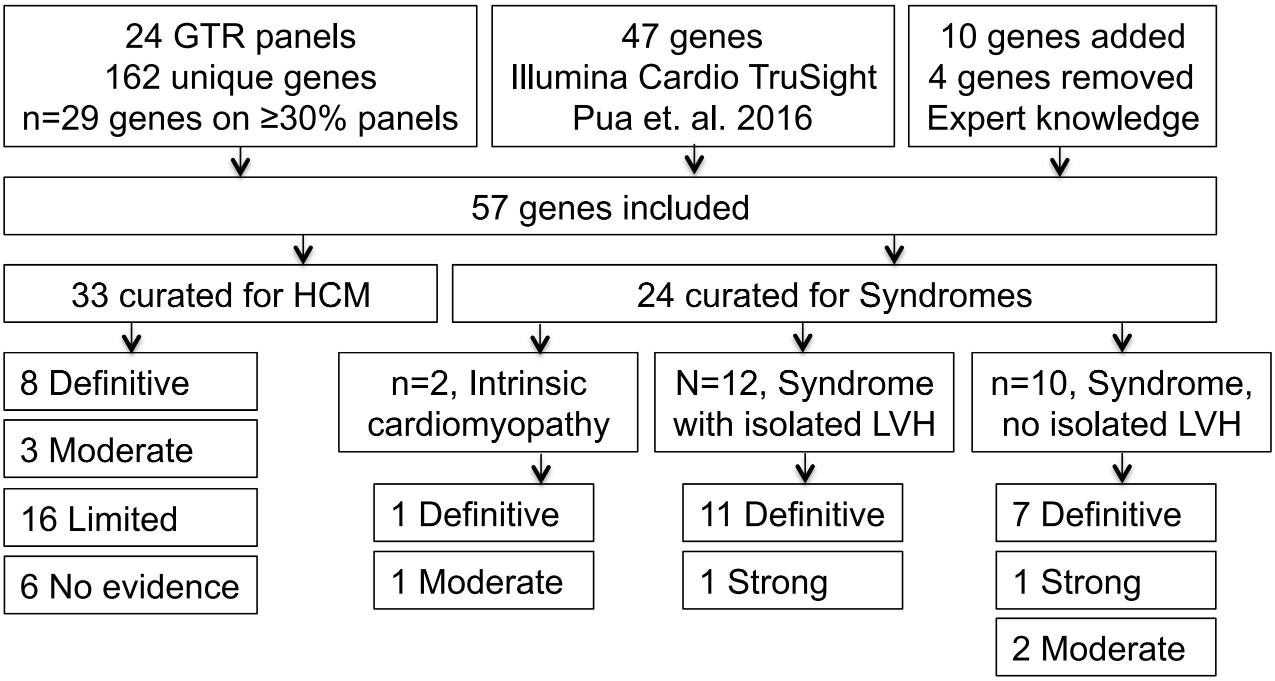

SUPPLEMENTARY FIGURE 3: Flowchart of the gene list and gene classifications

Abbreviations: GTR. Genetic testing registry; LVH, left ventricular hypertrophy

Written summaries of curations performed for Hypertrophic Cardiomyopathy (MonDO: 0005045)

***ACTA1*: Hypertrophic cardiomyopathy**
https://search.clinicalgenome.org/kb/gene-validity/10047
No convincing evidence for a causal role for *ACTA1* in hypertrophic cardiomyopathy (HCM) has been reported. Three heterozygous missense variants were found in probands with HCM secondary to nemaline myopathy or central core disease (Kaindl et al, 2004, PMID 15520409; D'Amico et al, 2006, PMID 16945537; Kim et al, 2011, PMID 21570694). There is no convincing case-control evidence for an association between *ACTA1* and HCM. Although this gene-disease association is supported by expression studies, no reports have convincingly implicated the gene in humans. **In summary, there is no reported evidence to support this gene-disease association with HCM.** This classification was approved by the Hypertrophic Cardiomyopathy Gene Curation Expert Panel on November 1, 2016.

***ACTC1*: Hypertrophic cardiomyopathy**
https://search.clinicalgenome.org/kb/gene-validity/8766
The *ACTC1* gene has been associated with autosomal dominant hypertrophic cardiomyopathy (HCM) in at least 6 probands in 4 publications. Four unique variants (missense) with convincing evidence of pathogenicity have been reported in humans, including *de novo* inheritance with maternity and paternity confirmed in two cases and segregation with disease in 26 additional family members. *ACTC1* was first associated with this disease in humans in 1999 (Mogensen et al, PMID 10330430). More evidence is available in the literature, but the maximum score for genetic evidence (12 pts.) has been reached. The mechanism for disease is unknown. The *ACTC1* gene was significantly enriched for missense variants in Walsh et al, 2016 (PMID 27532257). Overall, the gene was found to have an Odds Ratio of 8.59 (5.06-14.5) for HCM. This gene-disease association is supported by expression studies, *in vitro* functional assays, and an animal model. **In summary, *ACTC1* is definitively associated with autosomal dominant HCM.** This has been repeatedly demonstrated in both the research and clinical diagnostic settings, and has been upheld over time. This classification was approved by the ClinGen Hypertrophic Cardiomyopathy Expert Panel on September 5, 2017.

***ANKRD1*: Hypertrophic Cardiomyopathy**
https://search.clinicalgenome.org/kb/gene-validity/10043
The *ANKRD1* gene has been associated with autosomal dominant hypertrophic cardiomyopathy (HCM) in 5 probands in 2 publications. Three unique missense variants have been reported in humans with no family history of cardiomyopathy. *ANKRD1* was first associated with this disease in humans in 2009 (Arimura et al, 19608031). Evidence suggest a gain-of-function mechanism for variants in this gene (Crocini et al, 2013, PMID 23572067). This gene-disease association is supported by expression studies in mature rat cardiomyocytes and the examination of contraction parameters using engineered heart tissues. **In summary, there is limited evidence to support this gene-disease association.** Although more evidence is needed to support a causal role, no convincing evidence has emerged that contradicts the gene-disease association. This classification was approved by the ClinGen Hypertrophic Cardiomyopathy Gene Curation Expert Panel on September 19, 2017.

***CACNB2*: Hypertrophic cardiomyopathy**
https://search.clinicalgenome.org/kb/gene-validity/10044
No convincing evidence for a causal role for *CACNB2* in hypertrophic cardiomyopathy (HCM) has been reported. Although this gene-disease association is supported by expression in heart, no reports have directly implicated the gene in HCM. **In summary, there is no reported evidence to support this gene-**

disease association. This classification was approved by the ClinGen Hypertrophic Cardiomyopathy Gene Curation Expert Panel on June 6, 2017.

***CALR3*: Hypertrophic cardiomyopathy**
https://search.clinicalgenome.org/kb/gene-validity/fd8036e5-bf01-4b89-b4fc-94080d835d99--2018-08-06T13:14:27
The *CALR3* gene has been associated with hypertrophic cardiomyopathy (HCM) in 2 probands in 1 publication. Two unique heterozygous variants of unknown significance (missense), with no experimental evidence to support their pathogenicity, have been reported in humans (Chiu et al, 2007, PMID 17655857). One additional variant was reported in a proband with variants in a number of HCM-associated genes, possibly indicating a modifying role for *CALR3* as suggested by the authors (Botillo et al, 2016, PMID 26656175). The mechanism for disease is unknown. There is no experimental evidence to support the gene-disease association. **In summary, there is limited evidence to support this gene-disease association**. Although more evidence is needed to support a causal role, no convincing evidence has emerged that contradicts the gene-disease association. This classification was approved by the ClinGen Hypertrophic Cardiomyopathy Gene Curation Expert Panel on February 7, 2017.

***CASQ2*: Hypertrophic cardiomyopathy**
https://search.clinicalgenome.org/kb/gene-validity/10064
No convincing evidence for a causal role for *CASQ2* in hypertrophic cardiomyopathy (HCM) has been reported. Two heterozygous missense variants were curated but neither was clearly asserted to cause the disease (Chui et al, 2007, PMID 17655857; Glotov et al, 2015, PMID 25892673). There is no convincing case-control evidence for an association between *CASQ2* and HCM. Although this gene-disease association is supported by expression studies, no reports have convincingly implicated the gene in HCM. **In summary, there is no reported evidence to support this gene-disease association.** This classification was approved by the ClinGen Hypertrophic Cardiomyopathy Gene Curation Expert Panel on February 7, 2017.

***CSRP3*: Hypertrophic cardiomyopathy**
https://search.clinicalgenome.org/kb/gene-validity/10067
The *CSRP3* gene has been associated with hypertrophic cardiomyopathy (HCM) in over 15 probands in 6 publications. Three unique heterozygous missense variants with some evidence to support their pathogenicity, a frameshift variant, and two splice site variants have been reported in humans (Geier et al, 2003, PMID 12642359; Bos et al, 2008, PMID 16352453; Geier et al, 2008, PMID 18505755; Andersen et al, 2009, PMID 19035361; Santos et al, 2012, PMID 22429680; Lopes et al, 2015, PMID 26455666). Two of the missense variants had convincing segregation evidence (Geier et al, 2003, PMID 12642359; Geier et al, 2008, PMID 18505755). Multiple rare missense variants, with no functional evidence to support their pathogenicity, have also been identified. The mechanism for disease is unknown. The gene-disease association is supported by its biochemical function (Hoffmann et al, 2014, PMID 24934443) and protein interaction with Z-disc components alpha-actinin 2 and TCAP (Hayashi et al, 2004, PMID 15582318; Knoll et al, 2010, PMID 20044516) and expression data (Hoffmann et al, 2014, PMID 24934443). **In summary, there is moderate evidence to support this gene-disease association with HCM**. While more evidence is needed to establish this association definitively, no convincing contradictory evidence has emerged. This classification was approved by the ClinGen Hypertrophic Cardiomyopathy Gene Curation Expert Panel on December 19, 2017.

***DSP*: Hypertrophic cardiomyopathy**
https://search.clinicalgenome.org/kb/gene-validity/10065
A single missense variant (p.Gln647His) was detected in *DSP* in a patient with hypertrophic cardiomyopathy (HCM) who also harbored a *TTN* p. Asn9432Lys missense variant. The authors noted digenic HCM inheritance due to these two variants, and the 50 year-old proband had a grandfather who suffered sudden cardiac death below 40 years of age (Waldmüller et al, 2015, PMID 25979592). However, no assertions were made in the manuscript regarding whether *DSP* is associated with HCM, and it was not possible to determine the pathogenicity of this variant. **In summary, there is no reported evidence to support this gene-disease association.** This classification was approved by the ClinGen Hypertrophic Cardiomyopathy Gene Curation Expert Panel on May 23, 2017.

***JPH2*: Hypertrophic cardiomyopathy**
https://search.clinicalgenome.org/kb/gene-validity/8500
The *JPH2* gene has been associated with hypertrophic cardiomyopathy (HCM) in 7 probands in 4 publications. Six unique heterozygous variants (missense), with some evidence to support their pathogenicity, have been reported in humans (Landstrom et al, 2007, PMID 17509612; Beavers et al, 2013, PMID 23973696; Bongini et al, 2016, PMID 26869393; Quick et al, 2017, PMID 28393127). *JPH2* was first associated with this disease in humans in 2007 (Landstrom et al, PMID 17509612). The mechanism for disease is unknown. The gene-disease association is supported by expression studies, *in vitro* assays, and animal models. **In summary, there is moderate evidence to support this gene-disease association**. While more evidence is needed to establish this association definitively, no convincing contradictory evidence has emerged. This classification was approved by the ClinGen Hypertrophic Cardiomyopathy Gene Curation Expert Panel on July 18, 2017.

***KCNQ1*: Hypertrophic cardiomyopathy**
https://search.clinicalgenome.org/kb/gene-validity/10059
The KCNQ1 gene has been associated with autosomal dominant hypertrophic cardiomyopathy (HCM) using the ClinGen Clinical Validity Framework. This association was made using Case-level data alone. Only one 1 missense variant has been reported in an individual that presented with HCM and long QTc intervals (D'Argenio et al, 2014, PMID 24183960). Furthermore, the individual also harbored variants in two other genes associated with hypertrophic cardiomyopathy, *MYBPC3* (c.3627+2T>A) and *TNNT2* (c.459+175G>A). *MYBPC3* (c.3627+2T>A) is considered pathogenic and segregated in individuals in the family presenting with hypertrophic cardiomyopathy. While the D'Argenio paper asserts pathogenicity for the *KCNQ1* variant, it is unclear whether this assertion is for the the Long QT or HCM phenotypes observed in the proband. ***Furthermore, the current ClinGen Clinical Validity Framework is for Mendelian inheritance and does not support classifications for disease in which multiple genes may be contributory.*** Of note, this gene has also been implicated in Long QT syndrome, and this gene- disease relationship will be assessed separately. There is no experimental evidence to support the association at this time. In summary, there is **no reported evidence of Mendelian inheritance** to support this gene-disease association. Although more evidence is needed to support a causal role, no convincing evidence has emerged that contradicts or refutes the gene-disease association. This classification was approved by the ClinGen Hypertrophic Cardiomyopathy Expert Panel on January 4, 2017.

***KLF10*: Hypertrophic cardiomyopathy**
https://search.clinicalgenome.org/kb/gene-validity/10077
The *KLF10* gene has been associated with autosomal dominant hypertrophic cardiomyopathy using the ClinGen Clinical Validity Framework. This association was made using case-level data and case-control data. At least 5 unique missense variants have been reported, and an additional variant that is predicted

benign based on maximum allele frequency in the general population above the recommended cutoff for pathogenicity (Bos et al, 2012, PMID 22234868). *KLF10* was first associated with this disease in humans as early as 2012. The association was observed in only 6 probands from one publication (Bos et al, 2012 PMID 22234868). No segregation data is available. The mechanism for disease is unclear, but predicted to be loss of function (LOF) from functional assays performed by Bos et al, 2012. This gene-disease association is supported by expression studies showing enhanced expression of *KLF10* in skeletal muscle and heart (lesser expression in placenta and pancreas) (Subramaniam et al, 1995 PMID 8532536; Jiang et al, 2010, PMID 20201061), functional alterations in non-patient cells (Li et al, 2015 PMID 26252173), and an animal model (KLF10 knockout mice, Rajamannan et al, 2007, PMID 16888812). **In summary, there is limited evidence to support this gene-disease association.** Although more evidence is needed to support a causal role, no convincing evidence has emerged that contradicts the gene-disease association. This classification was approved by the ClinGen Hypertrophic Cardiomyopathy Expert Panel on August 1, 2017.

***MYBPC3*: Hypertrophic cardiomyopathy**
https://search.clinicalgenome.org/kb/gene-validity/10061
The *MYBPC3* gene has been associated with autosomal dominant hypertrophic cardiomyopathy (HCM) using the ClinGen Clinical Validity Framework. This association was made using case-level data and case-control data. *MYBPC3* was first associated with HCM in 1995 (Watkins et al, 1995, PMID 7493025). There are over 290 variants asserted as pathogenic for *MYBPC3* for HCM in ClinVar, and mutations in *MYBPC3* are reported in 40% of the reported cases of HCM (Cirino and Ho, 2014, GeneReviews, PMID 20301725). More evidence is available in the literature, but the maximum score for genetic evidence and/or experimental evidence (12 pts.) has been reached. Of note, *MYBPC3* has been shown to cause HCM in an autosomal recessive fashion, with earlier and more severe presentation of phenotypes associated with HCM, and represents a semi-dominant condition. The molecular mechanism for HCM is loss of function (LOF), and missense, nonsense, frameshift and splice site mutations in *MYBPC3* have been shown to be pathogenic for cardiomyopathy. Of note, this gene has been implicated in dilated cardiomyopathy and left ventricular noncompaction. This gene-disease association is supported by biochemical, expression, protein interaction, and animal models evidence. **In summary, *MYBPC3* is definitively associated with autosomal dominant HCM. This has been repeatedly demonstrated in both the research and clinical diagnostic settings, and has been upheld over time.** This classification was approved by the ClinGen Hypertrophic Cardiomyopathy Expert Panel on September 5, 2017.

MYH6: Hypertrophic cardiomyopathy
https://search.clinicalgenome.org/kb/gene-validity/10074
The *MYH6* gene has been associated with hypertrophic cardiomyopathy (HCM) in 3 probands in 2 publications. Three unique heterozygous variants of unknown significance (missense), with no experimental evidence to support their pathogenicity, have been reported in humans (Niimura et al 2002, PMID 11815426; Rubattu et al, 2016, PMID 27483260). Three additional variants were reported in patients with HCM (Carniel et al, 2005, PMID 15998695; Santos et al 2012, PMID 22429680) but, upon review, were considered too common to be disease-causing. The mechanism for disease is unknown. Experimental evidence to support the gene-disease association includes expression data (Gorza et al, 1984, PMID 6234108) and a biochemical function similar to a known HCM gene, MYH7 (unconventional myosin). **In summary, there is limited evidence to support this gene-disease association**. Although more evidence is needed to support a causal role, no convincing evidence has emerged that contradicts the gene-disease association. This classification was approved by the ClinGen Hypertrophic Cardiomyopathy Gene Curation Expert Panel on November 21, 2017.

***MYH7*: Hypertrophic cardiomyopathy**
https://search.clinicalgenome.org/kb/gene-validity/10075
Variation in the *MYH7* gene is associated with autosomal dominant hypertrophic cardiomyopathy (HCM). HCM was first linked to variation in *MYH7* in 1990 (Geisterfer-Lowrance et al, 1990, PMID 1975517). Over 100 missense pathogenic variants have subsequently been reported in humans with HCM (Alfares et al, 2015, PMID: 25611685), and robust association and/or linkage has been demonstrated for many distinct variants (Watkins et al, 1992, PMID: 1552912). Not all evidence in the literature has been curated, but the maximum score for genetic evidence (12 pts) is reached. The mechanism for disease is gain of function. This gene-disease association is supported by functional assays (including Han et al, 2014, PMID 25209314; Lan et al, 2013, PMID 23290139; Sommese et al, 2013, PMID 23798412), expression of *MYH7* in heart tissue (Perryman et al, 1992, PMID 1634614), and an HCM mouse model that recapitulates the phenotype (Geisterfer-Lowrance et al, 1996, PMID 8614836). **In summary, MYH7 is definitively associated with autosomal dominant HCM.** This has been repeatedly demonstrated in both the research and clinical diagnostic settings, and has been upheld over time. This classification was approved by the ClinGen Hypertrophic Cardiomyopathy Gene Curation Expert Panel on November 1, 2016.

***MYL2*: Hypertrophic Cardiomyopathy**
https://search.clinicalgenome.org/kb/gene-validity/8768
The *MYL2* gene has been associated with autosomal dominant hypertrophic cardiomyopathy (HCM) in at least 40 probands in 10 publications (Poetter et al, 1996, PMID 8673105; Flavigny et al, 1998, PMID 9535554; Kabaeva et al, 2002, PMID 12404107; Richard et al, 2003, PMID 12707239; Morner et al, 2003, PMID 12818575; Garcia-Pavia et al, 2011, PMID 21896538; Santos et al, 2012, PMID 22429680; Berge et al, 2013, PMID 24111713; Lopes et al, 2015, PMID 25351510; Claes et al, 2015, PMID 26497160). More than 8 unique variants (missense, splice site, nonsense, frameshift) have been identified in humans, and convincing segregation data has been reported (Flavigny et al, 1998, PMID 9535554). In addition, at least 8 missense VUSs in *MYL2* have been reported in patients with HCM. *MYL2* was first associated with HCM in humans in 1996 (Poetter et al, 1996, PMID 8673105). The *MYL2* gene was significantly enriched for missense variants in Walsh et al, 2016 (PMID 27532257) with and odds ratio of 6.74 (95% CI 4.69-9.70) for non-truncating variants and 3.10 (95% CI 0.89-10.7) for truncating variants. This gene-disease association is supported by expression data (Price et al, 1980, PMID 7236212), interaction with other known HCM gene products (MYH7, MYL3) (Rayment et al, 1993, PMID 8316857), and animal models (Szczesna-Cordary et al, 2005, PMID 16076902; Wang et al, 2006, 16837010; Kerrick et al, 2009, PMID 18987303). **In summary, *MYL2* is definitively associated with autosomal dominant HCM.** This has been repeatedly demonstrated in both the research and clinical diagnostic settings, and has been upheld over time. This classification was approved by the ClinGen Hypertrophic Cardiomyopathy Gene Curation Expert Panel on February 7th, 2017.

***MYL3*: Hypertrophic Cardiomyopathy**
https://search.clinicalgenome.org/kb/gene-validity/8499
The *MYL3* gene has been associated with autosomal dominant hypertrophic cardiomyopathy (HCM) in at least 20 probands in 13 publications. More than 8 unique variants (mostly missense, 1 splice acceptor variant) have been reported in humans, and variants in this gene segregated with disease in 24 additional family members. *MYL3* was first associated with HCM in humans in 1996 (Poetter et al, PMID 8673105). The *MYL3* gene was significantly enriched for missense variants in Walsh et al. 2016 (PMID 27532257), with an Odds Ratio of 5.00 (3.43-7.27) for HCM. This gene-disease association is supported by expression studies (Fujimoto et al, 1993, PMD 8417110), a mouse model (Vemuri et al, 1991, PMID 9927691), and evidence of interaction with *MYH7* (Petzhold et al, 2011, PMID 21262909) as well as

ACTC1 (Haase et al, 2006, PMID 16675844). **In summary, *MYL3* is definitively associated with autosomal dominant HCM.** This has been repeatedly demonstrated in both the research and clinical diagnostic settings, and has been upheld over time. This classification was approved by the ClinGen Hypertrophic Cardiomyopathy Gene Curation Expert Panel on December 22, 2017.

MYLK2: Hypertrophic cardiomyopathy
https://search.clinicalgenome.org/kb/gene-validity/10052
The MYLK2 gene has been associated with autosomal dominant hypertrophic cardiomyopathy using the ClinGen Clinical Validity Framework as of January 18, 2017. This association was made using Case-level data only. At least 2 unique missense variants have been reported in one individual *in cis* (Davis et al., 2001 PMID: 11733062). MYLK2 was first associated with this disease in humans as early as 2001. The association was observed in only 1 individual (Davis et al., 2001 PMID: 11733062), that also harbored a variant of unknown significance in MYH7 (p.E743D), a gene which is also implicated in the disease hypertrophic cardiomyopathy. No segregation data is available. The mechanism for disease is unknown, but predicted to be gain of function (GOF) from functional assays performed by Davis et al., 2001. This gene-disease association is supported by expression studies showing restricted expression of MYLK2 in skeletal muscle and heart, cell culture model system, and an animal model. **In summary, there is limited evidence to support this gene-disease association.** Although more evidence is needed to support a causal role, no convincing evidence has emerged that contradicts the gene-disease association. This classification was approved by the ClinGen Hypertrophic Cardiomyopathy Expert Panel on April 17, 2017.

***MYOM1*: Hypertrophic cardiomyopathy**
https://search.clinicalgenome.org/kb/gene-validity/8ff00fbf-6b20-49cd-8af8-0f6404240db5--2018-08-01T18:51:28
The *MYOM1* gene has been associated with hypertrophic cardiomyopathy (HCM) in 1 proband in 1 publication. A single missense variant, p.Val1490Ile, segregated in 2 affected family members with HCM and destabilized dimerization of the MYOM1 protein (Siegert et al, 2011, PMID 21256114). The mechanism for disease is unknown. This gene-disease association is supported by an expression study (Schoenauer et al, 2011, PMID 21069531). **In summary, there is limited evidence to support this gene-disease association**. Although more evidence is needed to support a causal role, no convincing evidence has emerged that contradicts the gene-disease association. This classification was approved by the ClinGen Hypertrophic Cardiomyopathy Gene Curation Expert Panel on July 20th, 2017.

***MYOZ2*: Hypertrophic cardiomyopathy**
https://search.clinicalgenome.org/kb/gene-validity/d74395ba-66ee-43c6-8d3c-c353bd8e5e82--2018-08-01T15:46:03
The *MYOZ2* gene has been associated with hypertrophic cardiomyopathy (HCM) in 5 probands in 3 publications. *MYOZ2* was first associated with this disease in humans in 2007 (Osio et al, PMID 17347475). One variant (missense), with experimental evidence to support its pathogenicity, was identified in a patient and was shown to segregate with 5 additional family members (Osio et al, 2007, PMID 17347475). Three additional unique variants with little to no experimental evidence to support their pathogenicity were reported (Osio et al, 2007, PMID 17347475; Posch et al, 2008; PMID 18591919). However, the frequencies of these variants in the ExAC database (exac.broadinstitute.org) are consistent with benign variation. Another unique variant (missense) was identified in a proband that also harbored a variant in *MYH7* and was excluded as causative (Guo et al, 2017, PMID 28296734). The mechanism for disease is unknown. The gene-disease association is supported by expression data, an interaction with *ACTN2*, and a mouse model. **In summary, there is limited evidence to support this**

gene-disease association. Although more evidence is needed to support a causal role, no convincing evidence has emerged that contradicts the gene-disease association. This classification was approved by the ClinGen Hypertrophic Cardiomyopathy Gene Curation Expert Panel on June 20, 2017.

***MYPN*: Hypertrophic cardiomyopathy**
https://search.clinicalgenome.org/kb/gene-validity/10048
The *MYPN* gene has been associated with hypertrophic cardiomyopathy (HCM) in 9 probands in 2 publications. *MYPN* was first associated with this disease in humans in 2010 (Bagnall et al, PMID 20801532). The proband identified in this paper was shown to also harbor a pathogenic variant in *MYH7*. Five unique variants of unknown significance (4 missense, 1 nonsense) with no experimental evidence to support their pathogenicity have been identified (Purevjav et al, 2012, PMID 22286171) in addition to 4 variants that are predicted to be benign. The mechanism for disease is unknown. The gene-disease association is supported by expression studies, a mouse model, and an *in vitro* assay. **In summary, there is limited evidence to support this gene-disease association**. Although more evidence is needed to support a causal role, no convincing evidence has emerged that contradicts the gene-disease association. This classification was approved by the ClinGen Hypertrophic Cardiomyopathy Gene Curation Expert Panel on November 1, 2016.

***NEXN*: Hypertrophic cardiomyopathy**
https://search.clinicalgenome.org/kb/gene-validity/8764
The *NEXN* gene has been associated with autosomal dominant hypertrophic cardiomyopathy in 2 probands in a single 2010 publication (Wang et al, PMID 20970104). Two unique missense variants were reported in this publication. The pathogenicity of one variant was supported by functional studies in a cell culture model system that showed mutant protein fragments accumulated in the cytoplasm and failed to bind to actin filaments. This variant also segregated with disease in 2 additional family members. The second variant reported by Wang et al. was found at a high frequency in the ExAC database, which is consistent with benign variation. The mechanism for disease is unknown. This gene-disease association is supported by a cell culture model system, expression studies and localization of NEXN to the intercalated disk (Hassel et al, 2009, PMID 1988149; Somi et al, 2016, 27148881), and interaction with alpha actin (Wang et al, PMID 20970104). **In summary, there is limited evidence to support this gene-disease association**. Although more evidence is needed to support a causal role, no convincing evidence has emerged that contradicts the gene-disease association. This classification was approved by the ClinGen Hypertrophic Cardiomyopathy Gene Curation Expert Panel, on December 22, 2017.

***OBSCN*: Hypertrophic cardiomyopathy**
https://search.clinicalgenome.org/kb/gene-validity/94265ca9-cf6a-468e-b12c-6ea173d09e51--2018-08-06T13:23:13
The *OBSCN* gene has been associated with hypertrophic cardiomyopathy (HCM) in 8 probands in 3 publications. Three unique heterozygous variants of unknown significance (1 missense, 2 frameshift) with no experimental evidence to support their pathogenicity have been reported in humans (Xu et al, 2015, PMID 26573135). *OBSCN* was first associated with this disease in humans in 2007 (Arimura et al, PMID 17716621. However, the frequencies of the variants reported in this publication and a variant reported in 2014 (Girolami et al, PMID 25173926) in the ExAC database (exac.broadinstitute.org) are consistent with benign variation. Three additional variants found in 3 unrelated probands in Xu et al, 2015 (PMID 26573135) were also excluded as causative after expert review. The mechanism for disease is unknown. The gene-disease association is supported by expression data in addition to an *in vitro* assay. **In summary, there is limited evidence to support this gene-disease association**. Although more

evidence is needed to support a causal role, no convincing evidence has emerged that contradicts the gene-disease association. This classification was approved by the ClinGen Hypertrophic Cardiomyopathy Gene Curation Expert Panel on February 1, 2017.

***PDLIM3*: Hypertrophic cardiomyopathy**
https://search.clinicalgenome.org/kb/gene-validity/76cba060-8080-42b1-b9ea-2193fea658b0--2018-08-06T13:21:24
The *PDLIM3* gene has been associated with HCM in 2 probands in 2 publications. One variant of unknown significance and 1 multi-exon deletion have been reported in humans with HCM. *PDLIM3* was first associated with HCM in humans in 2010 (Bagnall et al, 2010, PMID: 20801532). PDLIM is expressed in heart and interacts with ACTN2. However, there is no known mechanism through which PDLIM causes HCM. **In summary, there is limited evidence to support this gene-disease association with HCM**. Although more evidence is needed to support a causal role, no convincing evidence has emerged that contradicts the gene-disease association. This classification was approved by the ClinGen Hypertrophic Cardiomyopathy Gene Curation Expert Panel on November 1, 2016.

***RYR2*: Hypertrophic cardiomyopathy**
https://search.clinicalgenome.org/kb/gene-validity/10076
The *RYR2* gene has been associated with hypertrophic cardiomyopathy (HCM) in 4 probands in 2 publications. Four unique variants of unknown significance (3 missense, one splice variant) have been reported in humans with HCM. *RYR2* was first associated with HCM in humans in 2006 (Fujino et al, Circulation. 2006;114:II_165). The mechanism for disease is unknown. This gene-disease association is supported by expression studies. **In summary, there is limited evidence to support this gene-disease association.** Although more evidence is needed to support a causal role, no convincing evidence has emerged that contradicts the gene-disease association. This classification was approved by the ClinGen Hypertrophic Cardiomyopathy Gene Curation Expert Panel on February 7, 2017.

***TCAP*: Hypertrophic cardiomyopathy**
https://search.clinicalgenome.org/kb/gene-validity/8761
The *TCAP* gene has been associated with hypertrophic cardiomyopathy (HCM) in 7 probands in 3 publications. Six unique heterozygous variants of unknown significance (5 missense, 1 in-frame deletion), with no experimental evidence to support their pathogenicity, have been reported in humans (Hayashi et al, PMID 15582318; Bos et al, 2006, PMID 16352453; Andersen et al, 2009, PMID 19035361). *TCAP* was first associated with this disease in humans in 2004 (Hayashi et al, PMID 15582318). The frequencies of 2 variants in the ExAC database (exac.broadinstitute.org) are consistent with benign variation. Two probands were found to harbor disease-causing variants in the *TNNI3* gene and *MYBPC3* gene, respectively, in addition to a variant of unknown significance in the *TCAP* gene. The mechanism for disease is unknown. The gene-disease association is supported by expression data and an interaction with CSRP3. **In summary, there is limited evidence to support this gene-disease association**. Although more evidence is needed to support a causal role, no convincing evidence has emerged that contradicts the gene-disease association. This classification was approved by the ClinGen Hypertrophic Cardiomyopathy Gene Curation Expert Panel on November 1, 2016.

***TMPO*: Hypertrophic cardiomyopathy**
https://search.clinicalgenome.org/kb/gene-validity/a1e1bb21-c617-4aac-b9d8-1c2054d76c55--2018-08-01T17:19:30
No convincing evidence for a causal role for *TMPO* in hypertrophic cardiomyopathy (HCM) has been reported. One variant has been associated with dilated cardiomyopathy (Taylor et al, 2005, PMID

16247757), but was later disputed based on a review of the ExAC database (exac.broadinstitute.org). There is no convincing case control evidence for an association between *TMPO* and HCM. **In summary, there is no reported evidence to support this gene-disease association.** This classification was approved by the ClinGen Hypertrophic Cardiomyopathy Gene Curation Expert Panel on April 4, 2017.

***TNNC1*: Hypertrophic cardiomyopathy**
https://search.clinicalgenome.org/kb/gene-validity/8765
The *TNNC1* gene has been associated with hypertrophic cardiomyopathy (HCM) in 8 probands in 5 publications (Hoffman et al, 2001, PMID 11385718; Landstrom et al, 2008, PMID 18572189; Chung et al, 2011, PMID 21262074; Parvatiyar, 2012, PMID 22815480; Jaafar et al, 2015, PMID 26779504). Seven unique heterozygous variants (6 missense, 1 frameshift) with some evidence to support their pathogenicity and segregation in one family have been reported. *TNNC1* was first associated with this disease in humans in 2001 (Hoffman et al, PMID 11385718). The mechanism for disease is unknown. The gene-disease association is supported by *in vitro* functional studies, an animal model, and an interaction with TNNI3. **In summary, there is moderate evidence to support this gene-disease association.** Although more evidence is needed to establish this association definitively, no convincing contradictory evidence has emerged. This classification was approved by the ClinGen Hypertrophic Cardiomyopathy Gene Curation Expert Panel on November 1, 2016.

***TNNI3*: Hypertrophic cardiomyopathy**
https://search.clinicalgenome.org/kb/gene-validity/8769
The *TNNI3* gene has been associated with hypertrophic cardiomyopathy (HCM). *TNNI3* was first associated with this disease in humans in 1997 (Kimura et al, PMID 9241277). At least 60 unique variants, with varying levels of evidence to support their pathogenicity, have been reported in humans (reviewed in Mogensen et al, 2015, PMID 26440512). Variants in this gene segregated with disease in at least 6 families (Kimura et al, 1997, PMID 9241277; Rani et al, 2012, PMID 22876777; Choi et al, 2010, PMID 20641121; Mogensen et al, 2004, PMID 15607392). More evidence is available in the literature, but the maximum score for genetic evidence (12 pts) was reached. The mechanism for disease is likely dominant negative, as 91% of mutations reported are missense variants (Mogensen et al, 2015, PMID 26440512). The gene-disease association is supported by the function of the gene product, animal models, and in vitro assays**. In summary, *TNNI3* is definitively associated with hypertrophic cardiomyopathy.** This has been repeatedly demonstrated in both research and clinical diagnostic settings, and has been upheld over time. This classification was approved by the ClinGen Hypertrophic Cardiomyopathy Gene Curation Expert Panel on September 5, 2017.

***TNNT2*: Hypertrophic cardiomyopathy**
https://search.clinicalgenome.org/kb/gene-validity/8770
The *TNNT2* gene has been associated with hypertrophic cardiomyopathy. *TNNT2* was first associated with the disease in humans in 1994 (Thierfelder et al, 1994, PMID 8205619; Watkins et al, 1993, PMID 7981753; Watkins et al, 1995, PMID 7898523). Many unique variants, with varying levels of evidence to support their pathogenicity, have been reported in humans, as variants in *TNNT2* account for ~5% of HCM cases (Cirino and Ho, 2014, PMID 20301725). Variants in this gene segregated with disease in at least 3 families (Thierfelder et al, 1994, PMID8205619). More evidence is available in the literature, but the maximum score for genetic evidence (12 pts) was reached. The mechanism for disease is likely dominant negative, as most mutations reported are missense variants. The gene-disease association is supported by the function of the gene product, animal models, and *in vitro* assays. **In summary, *TNNT2* is definitively associated with hypertrophic cardiomyopathy.** This has been repeatedly demonstrated in both research and clinical diagnostic settings, and has been upheld over time. This classification was

approved by the ClinGen Hypertrophic Cardiomyopathy Gene Curation Expert Panel on November 7th, 2017.

***TPM1*: Hypertrophic cardiomyopathy**
https://search.clinicalgenome.org/kb/gene-validity/8771
The *TPM1* gene has been associated with hypertrophic cardiomyopathy (HCM). *TPM1* was first associated with this disease in humans in 1994 (Thierfelder et al, PMID 8205619). At least 15 unique heterozygous variants (missense), with varying levels of evidence to support their pathogenicity, have been reported in humans (reviewed in Redwood and Robinson, 2013, PMID 24005378). Variants in this gene segregated with disease in at least 7 families (Thierfelder et al, 1994, PMID 8205619; Jääskeläinen et al, 1998, PMID 9822100; Karibe et al, 2001, PMID 11136687; Jongbloed et al, 2003, PMID 12651045). More evidence is available in the literature, but the maximum score for genetic evidence (12 pts) has been reached. The mechanism for disease is likely dominant negative (Redwood and Robinson, 2013, PMID 24005378). The gene-disease association is supported the function of the gene product, animal models, and *in vitro* assays. **In summary, *TMP1* is definitively associated with HCM.** This has been repeatedly demonstrated in both research and clinical diagnostic settings, and has been upheld over time. This classification was approved by the ClinGen Hypertrophic Cardiomyopathy Gene Curation Expert Panel on December 20, 2016.

***TRIM63*: Hypertrophic cardiomyopathy**
https://search.clinicalgenome.org/kb/gene-validity/d9f35d3c-2d30-4947-9d10-87d21405586e--2018-08-01T16:33:16
The *TRIM63* gene has been associated with hypertrophic cardiomyopathy (HCM) in 3 probands in 1 publication. In another publication, it was associated with HCM and skeletal myopathy in 1 proband (Olivé et al, 2015, PMID 25801283). The authors reported a homozygous *TRIM63* p.Gln247X truncating variant as well as a *TRIM54* p.Asp106Asn missense variant within the proband, and interpreted these variants as pathogenic for his HCM and skeletal myopathy (Olivé et al, 2015, PMID 25801283). The same truncating variant (*TRIM63* p.Gln247X) was heterozygous in 2 unrelated probands with HCM, and a missense variant (p.Ile130Met) was found in an additional HCM proband (Chen et al, 2012, PMID 22821932). TRIM63 is expressed in heart and interacts with TNNI3 *in vitro*. However, there is no known mechanism through which *TRIM63* causes HCM. **In summary, there is limited evidence to support this gene-disease association**. Although more evidence is needed to support a causal role, no convincing evidence has emerged that contradicts the gene-disease association. This classification was approved by the ClinGen Hypertrophic Cardiomyopathy Gene Curation Expert Panel on December 20, 2016.

***TTN*: Hypertrophic cardiomyopathy**
https://search.clinicalgenome.org/kb/gene-validity/10077
The *TTN* gene has been associated with hypertrophic cardiomyopathy (HCM) in one family with an unusual presentation including variable dilatation, hypertrophy, and trabeculations with some family members meeting criteria for LVNC (Hastings et al, 2016, PMID 27625337). A heterozygous missense variant of unknown significance was reported in affected individuals in this family. Several other variants (missense, splice-site, in frame insertion) have been reported in *TTN* in patients with HCM (Satoh M et al, 1999, PMID 10462489; Arimura et al, 2009, PMID 19608031; Lopes et al, 2013, PMID 23396983; Waldmuller et al, 2015, PMID 25979592; Li et al, 2017, PMID 28223422). However, upon review, none of these variants were considered to have sufficient evidence to be disease-causing. The mechanism for disease is unknown. Experimental evidence to support the gene-disease association includes its biochemical function as a sarcomere component (Frazier et al, 2011, PMID 21297871) expression data (Uhlen et al, 2015, PMID 25613900), protein interaction with MYBPC3 (Freiburg A et al, 1996, PMID

8631348), functional alteration (Satoh M et al, 1999, PMID 10462489; Arimura et al, 2009, 19608031), and animal models (Peng J et al, 2007, PMID 17261657; Granzier et al, 2009, PMID 19679835). **In summary, there is limited evidence to support this gene-disease association**. Although more evidence is needed to support a causal role, no convincing evidence has emerged that contradicts the gene-disease association. This classification was approved by the ClinGen Hypertrophic Cardiomyopathy Gene Curation Expert Panel on December 14, 2017.

***VCL*: Hypertrophic cardiomyopathy**
https://search.clinicalgenome.org/kb/gene-validity/8762
The *VCL* gene has been associated with hypertrophic cardiomyopathy (HCM) in 2 probands in 2 publications. Two unique heterozygous variants of unknown significance (missense), with a limited amount of experimental evidence to support their pathogenicity, have been reported in humans (Vasile et al, 2006a, PMID 16236538; Vasile et al, 2006b, PMID 16712796). *VCL* was first associated with this disease in humans in 2006 (Vasile et al, PMID 16236538). The mechanism for disease is unknown. The gene-disease association is supported by expression data in addition to an *in vitro* functional assay**. In summary, there is limited evidence to support this gene-disease association.** Although more evidence is needed to support a causal role, no convincing evidence has emerged that contradicts the gene-disease association. This classification was approved by the ClinGen Hypertrophic Cardiomyopathy Gene Curation Expert Panel on October 18, 2016.

Written summaries of curations performed for Intrinsic Cardiomyopathy (MonDO: 0000591)

ACTN2: Intrinsic cardiomyopathy
https://search.clinicalgenome.org/kb/gene-validity/6710e329-d391-4355-85a5-02d03f8791a3--2018-08-06T13:12:55
ACTN2 was associated with hypertrophic cardiomyopathy in humans in 2006 (Theis et al, 2006, PMID 17097056) and dilated cardiomyopathy in 2010 (Zimmerman et al, 2010, PMID 20474083). Twelve unique heterozygous variants of unknown significance (11 missense, 1 frameshift), with little to no experimental evidence to support their pathogenicity, have been reported in *ACTN2* in humans. Missense variants in this gene segregated with atypical HCM in one family (max LOD 2.8) (Chiu et al, 2010, PMID 20022194), diverse cardiac phenotypes including sudden death, LVNC, DCM, and ventricular fibrillation in a second family (related to the first based on haplotype analysis) (Bagnal et al, 2014, PMID 25224718), and with combinations of HCM, LVNC and arrhythmia in another family (14 segregations) (Girolami et al, 2014, PMID 25173926). The mechanism for disease is unknown. Case-control studies have found no increased frequency of rare *ACTN2* variants in individuals with HCM, suggesting that much of the observed variation may be benign (Walsh et al, 2016, PMID 27532257). This gene-disease association is supported by the function of the protein (Bagnal et al, 2014, PMID 25224718), protein interaction with CSRP3 and SCN5A (Louis et al, 2007, PMID 9341203; Ziane et al, 2010, PMID 19943616; Vafiadaki et al, 2014, PMID 24860983), expression of the gene product (alpha actinin-2) in heart (Beggs et al, 1992, 1339456; Haywood et al, 2016, PMID 27287556), and rescue of a DCM phenotype in a zebrafish model (Gupta et al, 2012, PMID 22253474). **In summary, there is moderate evidence to support this gene-disease association**. While more evidence is needed to establish this association definitively, no convincing contradictory evidence has emerged. **Based on curated literature, this gene can cause seemingly isolated left ventricular hypertrophy.** Given the lack of constraint of *ACTN2*, classification of variants in this gene should be considered with care. This classification was approved by the ClinGen Hypertrophic Cardiomyopathy Gene Curation Expert Panel on November 7, 2017.

Lumping and Splitting: Per criteria outlined by the ClinGen Lumping and Splitting Working Group, we found no difference in the molecular mechanism underlying the disease entities: (1) Cardiomyopathy, dilated, 1AA, with or without LVNC (MIM: 612158) and (2) Cardiomyopathy, hypertrophic, 23, with or without LVNC (MIM: 612158). Note that both of these disease entities have the same MIM numbers, suggesting that they are part of the same condition. *ACTN2* was curated for intrinsic cardiomyopathy based upon the variable cardiac presentations, including cardiomyopathies and arrhythmias, observed in large families with *ACTN2* variants. For clinical management, all of the phenotypes are cardiovascular, and individuals should be monitored appropriately. Therefore, all of the disease entities have been lumped into one disease entity, ***Intrinsic cardiomyopathy.***

***PLN* : Intrinsic Cardiomyopathy**
https://search.clinicalgenome.org/kb/gene-validity/8772
The *PLN* gene is associated with hypertrophic cardiomyopathy, dilated cardiomyopathy, arrhythmogenic right ventricular cardiomyopathy (van der Zwaag, 2012 PMID: 22820313) and heart failure of unknown etiology. The typical inheritance for *PLN* related cardiomyopathy is autosomal dominant, albeit autosomal recessive inheritance has been noted and appears to follow a dosage effect or semidominance, as loss of both alleles results in an earlier and more severe phenotypic presentation (Haghighi, 2003 PMID: 12639993). *PLN* is encoded by one exon, and missense, nonsense, and frameshift mutations in the coding exon and the promoter region have been reported. Given that *PLN* only has one exon, many of the mutations (even LOF) result in a protein product. *PLN* encodes the 52 amino acid protein, phospholamban, that functions to regulate SERCA2 function in the sarcoplasmic reticulum. Phospholamban exists in both monomeric and homopentameric forms. The monomeric form is thought to inhibit SERCA2 activity. PLN-mediated SERCA2 inhibition is released upon phosphorylation of monomeric PLN by either PKA or CAMKII, and thus stabilization of the pentameric form (reviewed in Haghighi, 2014 PMID: 25451386, Young, 2015 PMID: 25563649). While the distinct genetic mechanism of *PLN*-mediated cardiomyopathy is unclear, the overall disease mechanism for *PLN* associated cardiomyopathy is dysregulation of SERCA2 function, Ca^{2+} handling, and disrupted relaxation and contraction of the heart. Multiple cases of *PLN*-mediated cardiomyopathy are reported in the literature, allowing and extending beyond the maximum score for genetic evidence (12 pts). This gene-disease association is supported by the function of the gene product, alteration of normal function in non-patient cells expressing patient-derived mutant *PLN*, and animal models. **In summary, *PLN* is definitively associated with intrinsic cardiomyopathy.** This association has been repeatedly demonstrated in both the research and clinical diagnostic settings, and has been upheld over time. **This classification was approved by the Hypertrophic Cardiomyopathy Gene Curation committee on September 19, 2017.**

Lumping and Splitting: Per criteria outlined by the ClinGen Lumping and Splitting Working Group, we found no difference in molecular mechanism(s) underlying the disease entities: (1) Cardiomyopathy, dilated, 1P (MIM: 609909) and (2) Cardiomyopathy, hypertrophic, 18 (MIM:613874). Evidence suggests that the mechanism of the disease is impaired SERCA2 regulation of Ca^{2+} handling for all conditions associated with *PLN*. Furthermore, a progressive cardiomyopathy beginning with hypertrophic and leading to dilated has been observed in a proband (Haghighi, 2003 PMID: 12639993). Both interfamilial and intrafamilial variability were observed between *PLN* variants (Haghighi, 2003 PMID: 12639993; van der Zwaag, 2012 PMID: 22820313). For clinical management, no striking differences should occur, as all of the phenotypes and conditions associated with *PLN* are of cardiovascular nature, and individuals should be monitored appropriately. Therefore, all of the disease entities have been lumped into one disease entity, ***Intrinsic cardiomyopathy.***

Data on the occurrence of cardiomyopathy in individuals with *PLN* associated intrinsic cardiomyopathy was collected by the Hypertrophic Cardiomyopathy Gene Curation Committee (subgroup of the Cardiovascular Working Group). Mutation of *PLN* results in the development of an intrinsic cardiomyopathy that can present with left ventricular hypertrophy. The mechanism for *PLN* induced intrinsic cardiomyopathy is attributed to SERCA2 dysfunction and improper Ca^{2+}- handling, which significantly alters contraction and relaxation of the heart, resulting in heart failure (reviewed in Haghighi, 2014 PMID: 25451386, Young, 2015 PMID: 25563649).

Gene Clinical Validity Curation Process

Standard Operating Procedure

Version 5

November 2017

The Clinical Genome Resource

Gene Curation Working Group

TABLE OF CONTENTS

BACKGROUND........ 3

REQUIRED COMPONENTS........ 3

OVERVIEW OF GENE CURATION........ 3

GENE CURATION WORKFLOW (Fig.1)........ 5

CLINICAL VALIDITY CLASSIFICATIONS (Fig. 2)........ 6

LITERATURE SEARCH........ 8

GENETIC EVIDENCE........ 10

- Case-level data........ 10
- Genetic Evidence Summary Matrix (Fig. 3)........ 11
  - Variant Evidence........ 13
  - Segregation analysis (Fig. 4-6)........ 15
- Case-control data........ 20
  - Examples (Fig. 7)........ 22

EXPERIMENTAL EVIDENCE........ 24

- Experimental Evidence Summary Matrix (Fig. 8)........ 24
  - Case-level vs Experimental Evidence........ 27

CONTRADICTORY EVIDENCE........ 28

SUMMARY & FINAL MATRIX (Fig. 9)........ 30

REFERENCES........ 32

BACKGROUND:

ClinGen's gene curation process is the method designed to aid in evaluating the strength of a gene-disease relationship based on publicly available evidence. Information about the gene-disease relationship, including genetic, experimental, and contradictory evidence curated from the literature is compiled and used to assign a clinical validity classification per criteria established by the ClinGen Gene Curation Working Group (GCWG)[1]. This protocol details the steps involved in curating a gene-disease relationship and subsequently assigning a clinical validity classification. This curation process is not intended to be a systematic review of all available literature for a given gene or condition, but instead an overview of the most pertinent evidence required to assign the appropriate clinical validity classification for a gene-disease relationship at a given time. While the following protocol provides guidance on the curation process, professional judgment must be used when deciding on the strength of different pieces of evidence that support a gene-disease relationship.

REQUIRED COMPONENTS:

- ClinGen-approved curation training. For training resources please see the ClinGen gene curation website (https://www.clinicalgenome.org/working-groups/gene-curation/ and https://www.clinicalgenome.org/curation-activities/gene-disease-validity/educational-and-training-materials/interactive-training-modules/) or contact clingen@clinicalgenome.org
- Internet browser
- Publication Access
- Access to the ClinGen Gene Curation Interface. https://curation.clinicalgenome.org/ Contact clingen-helpdesk@lists.stanford.edu for login information.

Optional: Microsoft Office (Word, Excel, or Powerpoint to record your data from curation)

OVERVIEW OF GENE CURATION:

The gene curation frame work consists of the following steps.

- **Collection of evidence:** The evidence is collected primarily from published peer-reviewed literature, but can also be present in publicly accessible resources, such as variant databases, which can be used with discretion. Literature searches can be conducted using PubMed (http://www.ncbi.nlm.nih.gov/pubmed) and/or Google Scholar

(http://scholar.google.com/) (which has a full-text search feature). Advanced searches are generally more informative.

  - PubMed tutorial: https://www.nlm.nih.gov/bsd/disted/pubmedtutorial/cover.html
  - PubMed presentation to ClinGen Biocurator working group: http://tinyurl.com/ydfd826m
  - Google Scholar search help: (https://scholar.google.com/intl/en/scholar/help.html#searching)

- One need not comprehensively curate all evidence for a gene-disease pair (particularly for "Definitive" associations), but instead focus on curating and evaluating the relevant pieces of evidence described in this protocol.
- **Identifying different evidence types:** The curator needs to identify and curate genetic and experimental evidence separately (details are defined later in "Genetic Evidence" and Experimental Evidence" sections). Genetic evidence is divided into two categories: case-level data and case-control data. Typically studies describing individuals or families with variants in the gene of interest will be scored as case-level data, while studies using statistical analysis to determine the enrichment of variants in case and control groups will be scored as case-control data. The gene-level experimental data used in this framework to assess a gene-disease relationship are *in vitro* and *in vivo* functional studies that implicate the causative role of a gene in disease. These are based on MacArthur and colleagues and described in detail below [2].
- **Assignment of clinical validity classification using gene curation matrix:** Next the curator evaluates the evidence and assigns points to the evidence using the scoring matrices provided below (Fig. 3,8). This information is then summarized and tallied to generate a total score and calculated clinical validity classification, which will be reviewed by a committee of appropriate disease experts.

Figure 1: Gene Curation Workflow

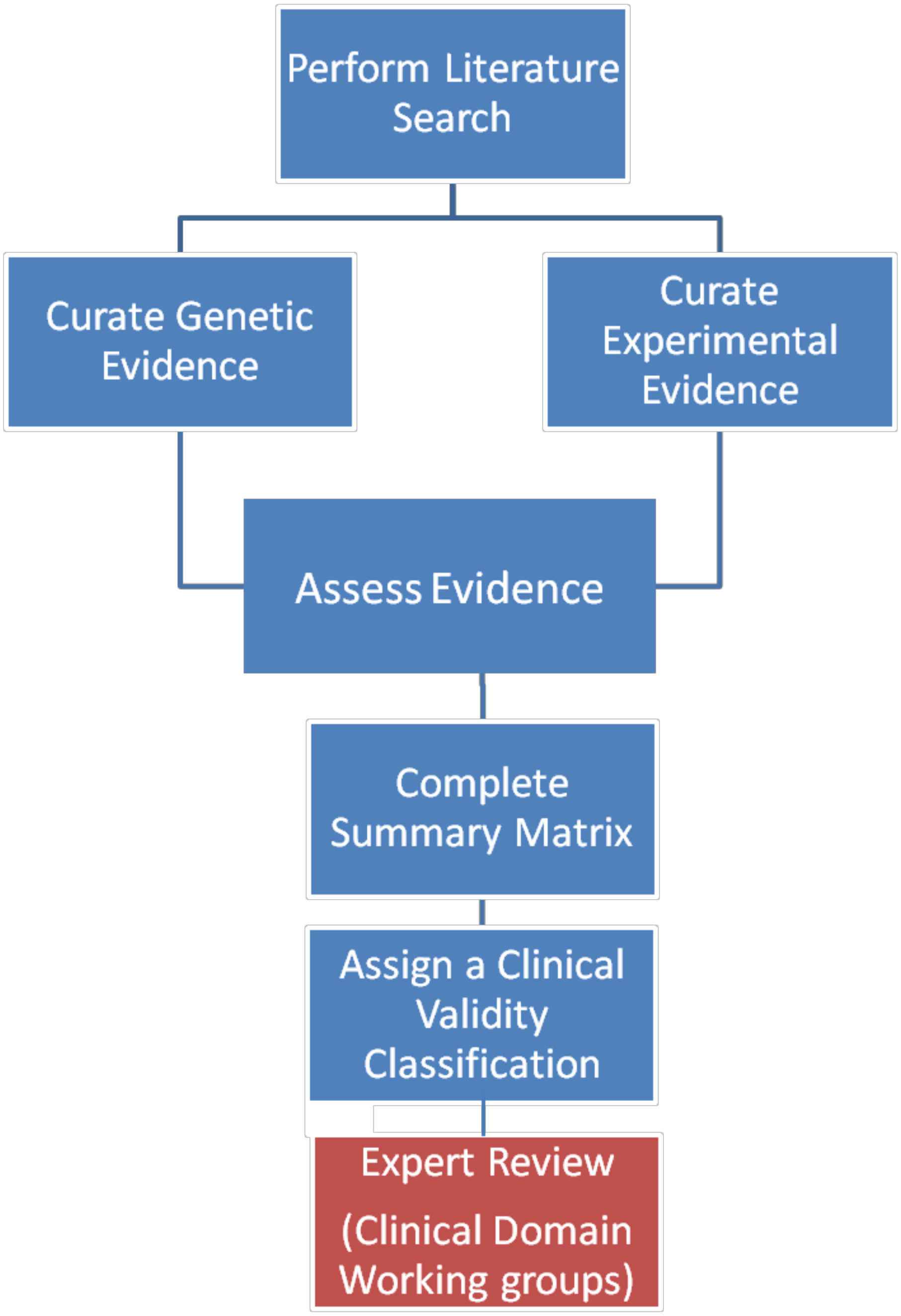

CLINICAL VALIDITY CLASSIFICATIONS:

The gene curation working group members have developed a method to qualitatively define the "clinical validity" of a gene-disease relationship using a classification scheme based on the strength of evidence that supports or refutes any claimed relationship. This framework allows the "clinical validity" of a gene-disease relationship to be transparently and systematically evaluated. These classifications can then be used to prioritize genes for analysis in various clinical contexts. The suggested minimum criteria needed to obtain a given classification are described for each clinical validity classification. These criteria include both genetic and experimental evidence, which are described below in this document. The default classification for genes without an identified variant in humans is "No Reported Evidence." The level of evidence needed for each supportive gene-disease relationship category builds upon the previous category (e.g. "Limited" builds upon "Moderate"). Gene-disease relationships classified as "Contradictory" likely have evidence supporting as well as opposing the gene-disease association, but are described separately from the classifications for supportive gene-disease relationships.

<table>
<tr><th colspan="2">Evidence Level</th><th>Figure 2: Clinical Validity Classifications (Evidence Description)</th></tr>
<tr><td rowspan="4">Supportive Evidence</td><td>DEFINITIVE</td><td>The role of this gene in this particular disease has been repeatedly demonstrated in both the research and clinical diagnostic settings, and has been upheld over time (in general, at least 3 years). No convincing evidence has emerged that contradicts the role of the gene in the specified disease.</td></tr>
<tr><td>STRONG</td><td>The role of this gene in disease has been independently demonstrated in at least two separate studies providing strong supporting evidence for this gene’s role in disease, including both of the following types of evidence:
• Strong variant-level evidence demonstrating numerous unrelated probands harboring variants with sufficient supporting evidence for disease causality[1]
• Compelling gene-level evidence from different types of supporting experimental data[2].
In addition, no convincing evidence has emerged that contradicts the role of the gene in the noted disease.</td></tr>
<tr><td>MODERATE</td><td>There is moderate evidence to support a causal role for this gene in this disease, including both of the following types of evidence:
• At least 3 unrelated probands harboring variants with sufficient supporting evidence for disease causality [1]
• Moderate experimental data[2] supporting the gene-disease association
The role of this gene in disease may not have been independently reported, but no convincing evidence has emerged that contradicts the role of the gene in the noted disease.</td></tr>
<tr><td>LIMITED</td><td>There is limited evidence to support a causal role for this gene in this disease, such as:
• Fewer than three observations of variants with sufficient supporting evidence for disease causality [1] OR
• Variants have been observed in probands, but none have sufficient evidence for disease causality.
• Limited experimental data[2] supporting the gene-disease association
The role of this gene in disease may not have been independently reported, but no convincing evidence has emerged that contradicts the role of the gene in the noted disease.</td></tr>
<tr><td colspan="2">NO REPORTED EVIDENCE</td><td>Evidence for a causal role in disease has not been reported. These genes might be “candidate” genes based on linkage intervals, animal models, implication in pathways known to be involved in human diseases, etc., but no reports have directly implicated the gene in human disease cases.</td></tr>
</table>

<table>
<tr><td>Contradictory Evidence</td><td>CONFLICTING EVIDENCE REPORTED</td><td>Although there has been an assertion of a gene-disease association, conflicting evidence for the role of this gene in disease has arisen since the time of the initial report indicating a disease association. Depending on the quantity and quality of evidence disputing the association, the association may be further defined by the following two sub-categories:
1. Disputed
a. Convincing evidence disputing a role for this gene in this disease has arisen since the initial report identifying an association between the gene and disease.
b. Disputing evidence need not outweigh existing evidence supporting the gene-disease association.
2. Refuted
a. Evidence refuting the role of the gene in the specified disease has been reported and significantly outweighs any evidence supporting the role.
b. This designation is to be applied at the discretion of clinical domain experts after thorough review of available evidence
c. While it is nearly impossible to entirely refute a gene’s potential role in disease, this category is to be used when all existing data has been fully refuted leaving the gene with essentially no valid evidence remaining, after an original claim.</td></tr>
<tr><td colspan="3">NOTES</td></tr>
<tr><td colspan="3">[1]Variants that disrupt function and/or have other strong genetic and population data (e.g. de novo occurrence, absence in controls, strong linkage to a small genomic interval, etc.) are considered convincing of disease causality in this framework. See "Variant Evidence" on p.13 for more information.
[2]Examples of appropriate types of supporting experimental data based on those outlined in MacArthur et al. 2014[2].</td></tr>
</table>

LITERATURE SEARCH:

Many human genes are implicated in more than one disorder. Therefore, prior to starting a curation and entering the details into the Gene Curation Interface, the curator should be absolutely clear on which disease entity is being curated. The expert group can give guidance if needed.

1. The initial search should be **broad** and **inclusive**. A good way to start is by searching **“gene symbol/name AND disease”** (in some cases it may be sufficient to search for the gene name/symbol alone). Ensure that you have looked up gene symbol/name alternatives before you search.
    a. Check HGNC (www.genenames.org/) for old gene symbols and aliases
    b. NCBI Gene (www.ncbi.nlm.nih.gov/gene) also lists gene aliases
    c. NOT all search results will be relevant, thus it is important to examine the search results for pertinent information

2. Curating primary literature is encouraged, but if a gene-disease pair has abundant information (i.e. >50 relevant results returned in a search), review articles may be sufficient. To find reviews, search PubMed with **“gene AND disease AND (review [Publication Type] OR "review literature as topic"[MeSH Terms]).**

   a. Curation may occur from that publication **ONLY** when sufficient details are included in the review article.
   b. If sufficient details are **NOT** included in the review article then the curator will need to return to each individual publication to curate the information.

3. Additional searches are often necessary to identify sufficient gene level experimental evidence. Note that additional gene level experimental evidence may exist in publications **BEFORE** the gene:disease association was first made.

   a. Search PubMed for experimental data (Examples below)
      - “gene AND function”
      - “protein AND function”
      - "gene AND animal”
   b. Additional information may also be available in OMIM (www.OMIM.org) in the **“Gene function"** or **“Biochemical Features”** sections
   c. GeneReviews (http://www.ncbi.nlm.nih.gov/books/NBK1116/) often has information in the **“Molecular Genetics”** section of the disease entries that may be useful.
   d. Other databases such as UniProt ([www.**uniprot**.org/](www.uniprot.org/)), MGI (www.informatics.jax.org/), etc. may also be useful, provided that primary references are given that can be curated. For a list of databases that may be helpful for the curation process, see Appendix A.
   e. GeneRIFs (Gene References Into Functions), within NCBI Gene, lists article links that summarize experimental evidence for a given gene. The link itself leads to an article in PubMed and can serve as an additional source for experimental evidence.

4. An additional component of the curation process is to determine if the original gene-disease association has been replicated; therefore, it is critical to find the **original paper** with the proposed relationship. OMIM and GeneReviews often cite the first publication and should be cross-referenced. Additionally, a recent review article may be helpful in ruling out any contradictory evidence that may have been reported since the original publication.

a. The "**Allelic Variants**" section of OMIM and the "**Molecular Genetics > Pathogenic allelic variants**" section of GeneReviews may have relevant information.
b. Be sure to extract information from the **original publication**, NOT directly from these websites.

Once all of the relevant literature about the gene-disease relationship has been assembled, curation of the different pieces of evidence can begin.

GENETIC EVIDENCE

Genetic evidence may be derived from **case-level data** (studies describing individuals or families with variants in the gene of interest) and/or **case-control data** (studies in which statistical analysis is used to evaluate enrichment of variants in cases compared to controls). While a single publication may include both case-level and case-control data, individual cases should NOT be double-counted (e.g., an individual case that is part of a case-control cohort should not be given points from both the "case-level data" and "case-control data" categories). **For example**, although this would be an unlikely situation, if a case from a case-control study were singled out and a pedigree was provided, this case could be evaluated with case-level data and segregations counted, but the case-control data itself should not be counted. In this scenario, a note should be made for expert review.

Genetic Evidence Summary Matrix

A matrix used to categorize and quantify the genetic evidence curated for a gene-disease pair is provided below. **NOTES:** All variants under consideration should be rare enough in the general population to be consistent with prevalence of disease.

Case-Level Data

Assessing case-level data requires knowledge of the inheritance pattern of the disease in question and careful interrogation of the individual variants identified in each case. Within this framework, a case should only be counted towards supporting evidence if the variant identified in that individual has some indication of a potential role in disease (e.g. impact on gene function, recurrence in affected individuals, etc.). Each case may be given points for both variant evidence (see below for details on interpretation) and segregation evidence (see p. 15 for details on calculation).

Figure 3: Genetic Evidence Matrix

<table>
<tr><th colspan="9">GENETIC EVIDENCE SUMMARY</th></tr>
<tr><th rowspan="2"></th><th colspan="2" rowspan="2">Evidence Type</th><th colspan="3" rowspan="2">Case Information</th><th colspan="2">Suggested Points/Case</th><th rowspan="2">Points Given</th><th rowspan="2">Max Score</th></tr>
<tr><th>Default</th><th>Range</th></tr>
<tr><td rowspan="9">Case-Level Data</td><td rowspan="5">Variant Evidence</td><td rowspan="3">Autosomal Dominant OR X-Linked Disorder A</td><td colspan="3">Variant is de novo</td><td>C 2</td><td>0-3</td><td>H</td><td>M 12</td></tr>
<tr><td colspan="3">Proband with predicted or proven null variant</td><td>D 1.5</td><td>0-2</td><td>I</td><td>N 10</td></tr>
<tr><td colspan="3">Proband with other variant type with some evidence of gene impact</td><td>E 0.5</td><td>0-1.5</td><td>J</td><td>O 7</td></tr>
<tr><td rowspan="2">Autosomal Recessive Disorder B</td><td colspan="3">Two variants in trans and at least one de novo or a predicted/proven null variant</td><td>F 2</td><td>0-3</td><td>K</td><td rowspan="2">P 12</td></tr>
<tr><td colspan="3">Two variants (not predicted/proven null) with some evidence of gene impact in trans</td><td>G 1</td><td>0-1.5</td><td>L</td></tr>
<tr><td colspan="2" rowspan="4">Segregation Evidence</td><td rowspan="4">Evidence of segregation in one or more families</td><td>Total LOD Score</td><td>Sequencing Method: Candidate Gene Sequencing</td><td>Sequencing Method: Exome/ Genome or all genes sequenced in linkage region</td><td rowspan="4">0-3</td><td rowspan="4">Q</td><td rowspan="4">R 3</td></tr>
<tr><td>2-2.99</td><td>0.5</td><td>1</td></tr>
<tr><td>3-4.99</td><td>1</td><td>2</td></tr>
<tr><td>≥5</td><td>1.5</td><td>3</td></tr>
<tr><td rowspan="3">Case-Control Data</td><td colspan="2">Case-Control Study Type</td><td colspan="3">Case-Control Quality Criteria</td><td colspan="2">Suggested Points/Study</td><td>Points Given</td><td>Max Score</td></tr>
<tr><td colspan="2">Single Variant Analysis</td><td colspan="3" rowspan="2">• Variant Detection Methodology
• Power
• Bias and Confounding Factors
• Statistical Significance</td><td colspan="2">0-6</td><td rowspan="2">S</td><td rowspan="2">T 12</td></tr>
<tr><td colspan="2">Aggregate Variant Analysis</td><td colspan="2">0-6</td></tr>
<tr><td colspan="9">TOTAL ALLOWABLE POINTS for Genetic Evidence</td><td>U 12</td></tr>
</table>

General Notes for variant scoring:

1. When curating an autosomal dominant disease or an X-linked disorder consider the evidence types in row "A". If you are curating an autosomal recessive disease, consider the evidence types in row "B". In X-linked disorders, affected probands will often be hemizygous males and/or manifesting heterozygous females. Recognizing that there can be rare cases of females affected by X-linked recessive disorders (due to chromosomal aneuploidy, skewed X inactivation, or homozygosity for a sequence variant), or males who carry an X-linked variant but are unaffected or mildly affected (due to Klinefelter syndrome, 47, XXY) evaluators must be aware of the nuances of interpretation of individual cases and X-linked pedigrees. Points can be assigned at the discretion of the expert reviewer taking into account the available evidence. Furthermore, there are known cases of female carriers of X-linked recessive conditions manifesting symptoms that are milder or later in onset compared to males, and scoring of genetic evidence in these examples should be subject to expert review with regard to the assigned gene/disease/inheritance combination.
2. Computational scores (such as conservation scores, constraint scores, *in silico* prediction tools, variation intolerance scores, etc) are often disease and context-dependent and should not be considered as strong pieces of evidence for variant pathogenicity. However, they can be recorded during curation and used as supporting evidence for variant scoring to be confirmed by expert review.
3. For a variant to be considered potentially disease-causing, its frequency in the general population should be consistent with phenotype frequency, inheritance pattern, disease penetrance, and disease mechanism (if known). These pieces of information can often be located in the literature (See "Literature Search" p. 8), but may also be contributed by experts. If such information is available, the prevalence of the variant in affected individuals should be enriched compared to controls. The Genome Aggregation Database (gnomAD; http://gnomad.broadinstitute.org) provides a reference set of allele frequencies for various populations and can be used to assess whether the frequency of the variant in question is consistent with the prevalence of the disease. Gene curation committees may find it helpful to set a maximum allele frequency (MAF) above which a variant would be considered benign. Generally, MAF thresholds will vary as a function of disease prevalence. This MAF threshold is specific to the disease and should apply to all variants being evaluated, in the context of that disease.
4. For each case information category, a suggested number of points per case is provided. However, the points may be altered, within a defined a range, to

account for the strength of evidence available to indicate that a variant is deleterious (see Figure 3). Within each range, the curator may choose one of the following scores: 0.1, 0.25, 0.5, followed by 0.5 point increments up to the maximum possible score for that category. However, the curator should always document reasons for any deviation in suggested scores for expert review.

5. When scoring variants for autosomal recessive disorders in individuals who are compound heterozygotes, there should be some evidence to suggest that the variants are in *trans* in order to be scored. For example, for an individual who is compound heterozygous for two variants in the gene of interest, both parents should be tested to show that the variants are in *trans*. Molecular methods showing that variants are in *trans* are also acceptable. For individuals who appear to be homozygous for a variant, testing of the parents is not required in order to count the case.

Variant Evidence:

1. Other variant with gene impact (Missense variants, small in-frame indels, etc.):
   a. Some functional impact to the gene product must be demonstrated for the case to be given default points. Examples of functional impact include reduced activity of an enzyme in cells expressing a variant in that gene, or reduced expression of a gene product in cells from an individual with a variant(s) in the gene. Impact based on functional validation can score 0.5 or above (up to 1.5/case) depending on the validation quality and disease relevance of the functional assay.
   b. *In silico* predictions do not provide strong evidence for functional impact and therefore, impact based on *in silico* predictions only would score less than the default 0.5 points. It may be appropriate to award default points if in-depth *in silico* modeling studies e.g. based on impact on 3D structure, have been done, but this requires discussion with an expert.
   c. Sum up the number of points. The suggested points per case can be found in column "E" (dominant) and "G" (recessive). Total up all of the variant evidence points and place them in "J" (dominant) or "L" (recessive), as appropriate.
2. Predicted or observed null variants

   Some types of variants can be assumed to disrupt gene function. This category includes nonsense, frameshift, canonical +/-1 or 2 splice site variants, single or multi-exon deletion, whole gene deletion, etc). For missense and small in frame insertions and deletions, see #1:
   a. Assign fewer points if there is alternative splicing or if the null variant is near the C terminus and/or nonsense medicated decay (NMD) is not

predicted (NOTE: NMD is not expected to occur if the stop codon is downstream of the last 50 bp of the penultimate exon).

b. Consider assigning fewer points if a gene product is still made, albeit altered. For example, cDNA analysis and Western blot for an individual with a canonical splice site change show that an exon is skipped but that the reading frame is maintained and a protein is produced.
c. Gene constraint scores can be helpful when assessing disease mechanism. For example, the disease mechanism could be assumed to be loss of function (LOF) if the gene is LOF constrained. Constraint scores can be found by searching the gene in ExAC (exac.broadinstitute.org) and viewing the "constraint metric" at the top right of the page. The closer the probability of LOF intolerance (pLI) is to 1, the more LOF-constrained the gene. However, constraint scores must be interpreted in the context of the gene and disease in question. For example, if the gene is associated with multiple diseases, LOF constraint could be associated with a disease other than the one being curated. In addition, genes associated with severe, pediatric-onset disorders may appear to be more constrained than adult-onset conditions where overall fitness is not impacted.
d. Individuals with large deletions, duplications, and other chromosomal rearrangements encompassing genetic material outside the gene of interest should not be counted because the impact of the loss/gain for the additional material cannot be assessed.
e. Sum up the number of points. The suggested points per case can be found in column "D". Total up all of the variant evidence points and place them in "I".

3. *De novo* variants:

a. These can be any type of variant, but should be given points depending on statistical expectation of *de novo* variation in the gene in question, if known. In some cases, this can be found in the literature and should be noted if found (See "literature search" p. 8). However, the curator may also leave this to be supplied by experts during curation review.
b. In order for a variant to be considered *de novo*, both parents must be tested to show that they do not carry the variant. Consider awarding default points to null variants (e.g. nonsense, frameshift, canonical splice site) that appear to be *de novo* based on testing parents for the variant, but award fewer points to missense variants and small, in-frame deletions. The scores can be increased if the maternity and paternity of the proband are confirmed e.g. by short tandem repeat analysis or trio whole exome sequencing (WES). For example, a case with a missense variant could receive default points if maternity and paternity are confirmed. Additional

points can be added for any variant if functional evidence supports a deleterious impact for the variant.

c. Sum up the number of points. The suggested points per case can be found in column "C". Total up all of the variant evidence points and place them in "H".

NOTE: In addition to meeting the above criteria, the variant should not have data that contradicts a pathogenic role, such as an unexplained non-segregation, etc. If the points given above for the summary matrix exceed the max score, use the Max score found in "M-P" for the summary matrix.

Segregation Analysis:

The use of segregation studies in which family members are genotyped to determine if a variant co-segregates with disease can be a powerful piece of evidence to support a gene-disease relationship.

For the purposes of this framework, we are employing a simplified analysis in which we assume the recombination fraction (θ) is zero (i.e. non-recombinants are not observed) to estimate a LOD score (see equations below). We suggest awarding different amounts of points depending on the methods used to investigate the linkage interval. For that reason, it is critical that the curator make a note of testing methodologies in families counted towards the segregation score. See below for a) instructions how to count segregations and calculate a simplified LOD score and b) how to evaluate the sequencing methods for the linkage interval and award points accordingly. Note that these are general guidelines; if you encounter cases where you are unsure how to evaluate/score segregation, please discuss with your expert group and/or the ClinGen Gene Curation working group.

Counting Segregations and Calculating Simplified LOD Scores:

<u>If a LOD score has been calculated by the authors of a paper:</u>
This LOD score should be documented and may be used to assign segregation points (according to the sequencing methods used to investigate the linkage region and identify the variants) in the scoring matrix (see Fig 6 for scoring suggestions). If a LOD score is provided by the authors, the ClinGen curator should not use the formula(s) below to estimate a new LOD score. If for some reason you do not agree with the published LOD score, do not assign any points and discuss the concerns with the expert reviewers. See below for more guidance on scoring. Fill out the "Segregation evidence" portion of the matrix. The number of points should be recorded in "Q".

<u>If a LOD score has NOT been calculated by the authors of a paper:</u>
Curators may estimate a LOD score using the simplified formula(s) below if the following conditions are met:

- The disorder is rare and highly penetrant.

o Phenocopies are rare or absent.

o For **dominant or X-linked disorders**, the estimated LOD score should be calculated using ONLY **families with 4 or more segregations present**. The affected individuals may be within the same generation, or across multiple generations.

o For **recessive disorders**, the estimated LOD score should be calculated using **ONLY families with at least 3 affected individuals in the pedigree**, including the proband). Genotypes must be specified for all affected and unaffected individuals counted; specifically, parents of affected individuals must be genotyped or other methods must be used to show that the variants are in *trans* if the affected individuals are noted to be compound heterozygotes.

o Families included in the calculation must not demonstrate any non-explainable non-segregations (for example, a genotype-/phenotype+ individual in a family affected by a disorder with no known phenocopies). Families with non-explainable non-segregations should not be used in LOD score calculations.

If any of the previous conditions are not met, do not use the formula(s) below to estimate a LOD score.

To be conservative in our simplified LOD score estimations, for autosomal dominant or X-linked disorders, only affected individuals (genotype+/phenotype+ individuals) or obligate carriers (regardless of phenotype) should be included in calculations. An obligate carrier is an individual who has not been tested for the variant in question but who is inferred to carry the variant by virtue of their position in the pedigree (for example, an individual with a parent with the variant and a child with the variant, an individual with a sibling with the variant and a child with the variant, etc.).

Within a given gene-disease curation, if more than one family meets the criteria above for scoring segregation information, sum their LOD scores to score (using the tables in Figures 4 or 5). For example, if Family A has an estimated LOD score of 1.2 and Family B has an estimated LOD score of 1.8, the summed LOD score will equal 3. See the discussion on sequencing method below for guidance on assigning points to the LOD score.

Expert reviewers may choose to specify the most appropriate way to approach segregation scoring within their disease domain, including enacting more formal, rigorous LOD score calculations.

NOTE: Segregation implicates a locus in a disease, NOT a variant. Therefore, all linkage studies should be carefully assessed to ensure that appropriate measures have been taken to rule out other possible causative genes within the critical region (see guide on point assignment based on methods to investigate a linkage region below).

For dominant/X-linked diseases:

$$Z \text{ (LOD score)} = \log_{10} \frac{1}{(0.5)^{\text{Segregations}}}$$

Figure 4: Dominant/X-linked LOD score table

Dominant Segregations	15	14	13	12	11	10	9	8	7	6	5	4
**Estimated LOD**	4.5	4.2	3.9	3.6	3.3	3.0	2.7	2.4	2.1	1.8	1.5	1.2

For recessive diseases:

$$Z\text{ (LOD score)} = \log_{10}\frac{1}{(0.25)^{\#\text{ of Affected Individuals-1}}\,(0.75)^{\#\text{ of Unaffected Individuals}}}$$

NOTE: In general, the number of affected individuals - 1 is equal to the number of affected segregations from the proband and can be used interchangeably in this equation. The "0.25" and "0.75" numbers used in this equation represent the risk of being affected vs. unaffected in a classic AR disease model in which both parents are carriers; if a particular pedigree differs (for example, parent has AR disorder in question and other parent is a carrier), please adjust those numbers to reflect the risk of inheritance.

Figure 5: Recessive LOD score table

		**Unaffecteds**										
		**0**	**1**	**2**	**3**	**4**	**5**	**6**	**7**	**8**	**9**	**10**
**Affecteds**	**3**	1.20	1.32	1.45	1.50	1.70	1.82	1.95	2.07	2.20	2.33	2.45
	**4**	1.81	1.93	2.06	2.18	2.31	2.43	2.56	2.68	2.81	2.93	3.06
	**5**	2.41	2.53	2.66	2.78	2.91	3.03	3.16	3.28	3.41	3.53	3.66
	**6**	3.01	3.14	3.26	3.39	3.51	3.63	3.76	3.88	4.01	4.13	4.26
	**7**	3.61	3.74	3.86	3.99	4.11	4.24	4.36	4.49	4.61	4.74	4.86
	**8**	4.21	4.34	4.46	4.59	4.71	4.84	4.96	5.09	5.21	5.34	5.46
	**9**	4.82	4.94	5.07	5.19	5.32	5.44	5.57	5.69	5.82	5.94	6.07
	**10**	5.42	5.54	5.67	5.79	5.92	6.04	6.17	6.29	6.42	6.54	6.67

Counting Segregations

1. In general, the number of segregations in the family will be the number of affected individuals minus one, the proband, to account for the proband's genotype phase being unknown. However, as there may be exceptions, segregations should be counted carefully, as outlined below.

For example, **pedigree A** shows a family with hypertrophic cardiomyopathy.

a. There are **four segregations** that can be counted beginning at the proband. This includes the mother (II-2) who is an obligate carrier and can be assumed to be genotype-positive even though she was not tested. Using **four segregations** in the formula above results in an estimated **LOD score of 1.2**.

b. For disorders with reduced penetrance such as cardiomyopathy, it is **safest to only use affected genotype+ individuals for segregation.** Obligate carriers (i.e. any

individual who can be definitively inferred to be genotype positive based on the genetic status of other family members, as discussed above) should also be included, regardless of phenotype. In this case, the absence of a phenotype in two genotype+ individuals (III-2 and III-5) is considered irrelevant as they can be explained by delayed onset and/or reduced penetrance. However, these individuals are not included in the calculation because they are unaffected.

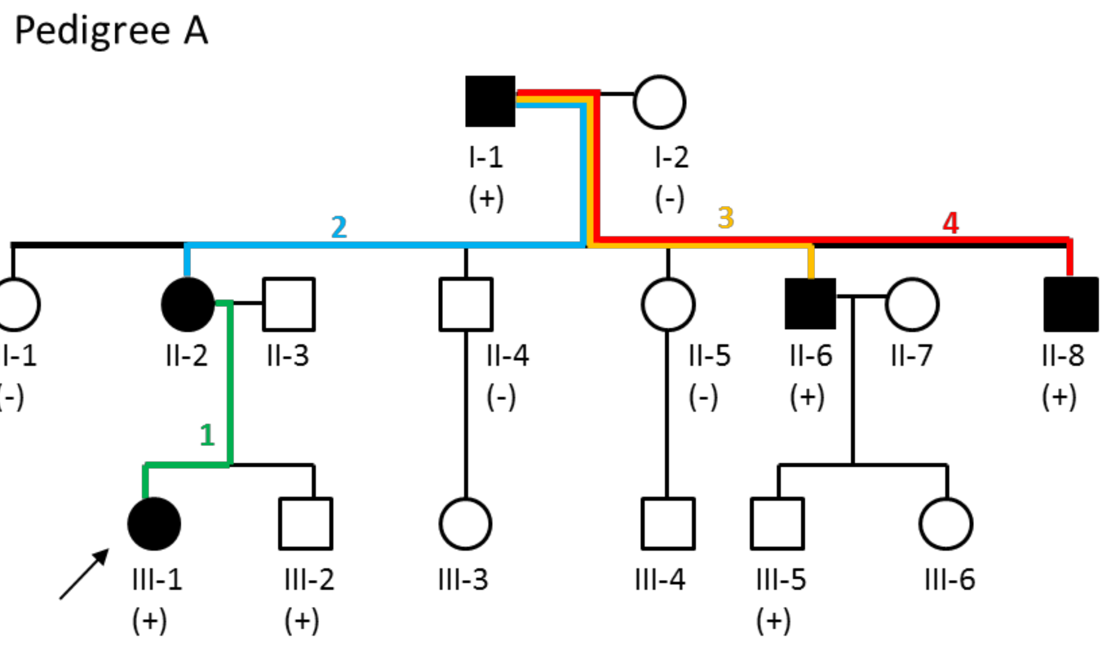

2. When estimating LOD scores for autosomal recessive disorders, count unaffected individuals as those who would be at the same risk to inherit two altered alleles as an affected individual, i.e. homozygous normal or heterozygous carrier siblings of a proband. For example, there are two unaffected individuals in Pedigree B, one unaffected individual in Pedigree C, and two unaffected individuals in Pedigree D.

3. For reasonably penetrant Mendelian disorders, a single LOD score can be calculated across multiple families, providing that each family meets the criteria above.

For example, in pedigrees B, C and D, each with fully penetrant recessive hearing loss, the LOD scores can be added ((1.5 for B) + (1.3 for C) + (1.5 for D)) to give a total LOD score of 4.3. However, pedigree E cannot be included in this LOD score total because this family does not have enough affected individuals.

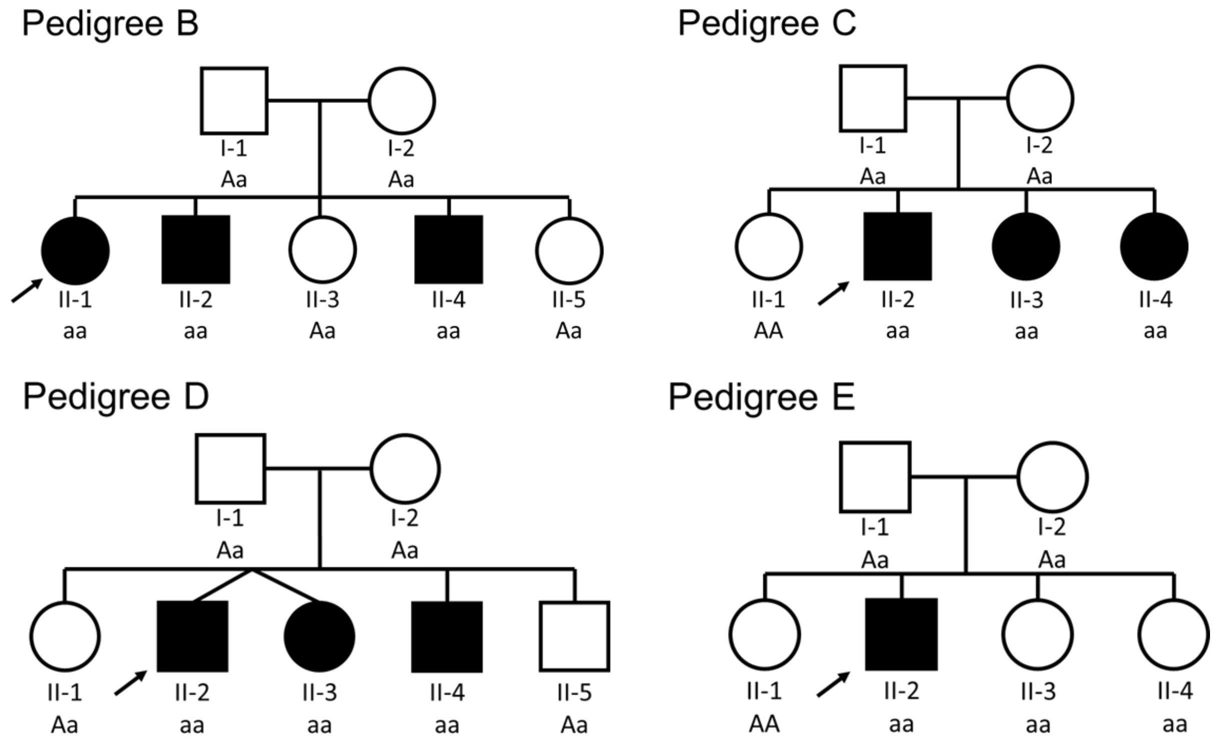

Assigning points to LOD scores:
While segregation evidence can be convincing for a particular locus, 10s or even 100s of genes can be within a linkage interval. Thus, segregation does not necessarily implicate a single gene or variant. Many publications do not thoroughly investigate other genes or variants found in the linkage interval and those that do cannot rule out the effects of thousands of other variants in the interval. **Thus, it is critical for a curator to evaluate the methods used to identify candidate variants.**
Some publications more thoroughly investigate the genes and variants in a linkage interval than others. Accordingly, more points are awarded for segregation evidence in cases where whole exome/genome sequencing was performed or if the entire linkage interval was sequenced. These methods provide more convincing evidence than a candidate gene approach in which only one or a handful of genes in a linkage region are sequenced. See figure 6 below for suggested point ranges for LOD scores.

NOTE: For this scoring matrix, LOD scores from all families meeting size requirements **must be summed before awarding segregation points, regardless of the sequencing methodology used**. Sequencing methodology (e.g., candidate gene sequencing, whole exome sequencing, etc.) should be taken into account when deciding on the most appropriate score for this evidence. See example 2 below for an example of scoring multiple families with variants ascertained via different methodologies. Note that simply having a single family meeting the minimum size requirements is not necessarily enough to warrant any points. As the methods in each publication vary, the suggested points in figure 6 are merely a guide for the curator.

Figure 6: Proposed Matrix Scoring for different LOD score ranges

Total summed LOD score across all families	Sequencing method	
	Candidate gene sequencing	Exome/genome or all genes sequenced in linkage region
0-1.99	0 pts	0 pts
2-2.99	0.5 pts	1 pts
3 - 4.99	1 pts	2 pts
(>/=) 5	1.5 pts	3 pts

Example Scenarios and Suggested Scoring:

Example 1: Linkage analysis was performed on one large family with autosomal dominant hypertrophic cardiomyopathy (HCM). There are **11 affected individuals** in the pedigree (phenotype +, genotype +), and using our simplified LOD score formula, this corresponds to a **LOD score of 3** (see Figure 4). The linkage region for this family contained 15 genes and the authors **sequenced all of the genes in the linkage interval** and the HCM variant was the only suspicious variant. Looking at Figure 6, you can award this LOD score **2 points**.

Example 2: Let's return to Pedigrees B, C, and D above, assuming now that we know more about how the linkage intervals were investigated or how the variants were identified.
Pedigree B: LOD Score 1.5, Variants identified using whole exome sequencing
Pedigree C: LOD Score 1.3, Variants identified using whole exome sequencing
Pedigree D: LOD Score 1.5, Variants identified using candidate gene analysis. Only the gene of interest was sequenced.
First, **we would sum the LOD scores across families**, which gives us a **LOD score of 4.3**. Because the variants were detected using **two different methods**, we can **opt to split the difference between the suggested point values** of 1 for candidate gene sequencing and 2 for whole exome sequencing and award this segregation analysis **1.5 points**.

We recognize that the methods in each publication vary, therefore the suggested points in figure 6 are merely a guide for the curator. If curators are unsure of segregation scoring based on genotyping method, please consult experts.

Case-Control Data:

Case-Control studies are those in which statistical analysis is used to evaluate enrichment of **variants in cases compared to controls**. Each case-control study should be independently assessed based on the criteria outlined in this section to evaluate the quality of the study design. Consensus with a clinical domain expert group is highly recommended.

1. Case-control studies are classified based on how the study is designed to evaluate variation in cases and controls: **single variant analysis** or **aggregate variant analysis**.
    a. **Single variant analysis studies** are those in which individual variants are evaluated for statistical enrichment in cases compared to controls. More than one variant may be analyzed, but the variants should be independently assessed with appropriate statistical correction for multiple testing. **For example**, if a study identifies 2 different variants in *MYH7* within a cohort of hypertrophic cardiomyopathy cases, but tests the number of hypertrophic cardiomyopathy cases and unaffected controls that contain only one of the variants and provides a statistic for that variant alone, then the study is classified as a single variant analysis. Similarly, if the same study tests for enrichment of the second variant in the cases and controls and provides a separate statistic for the second variant, this also is a single variant analysis. Often, authors will indicate this either in the article text or in a table of variants.
    b. **Aggregate variant analysis** studies are those in which the statistical enrichment of two or more variants as an aggregate is assessed in cases compared to controls. This comparison could be accomplished by genotyping specific variants or by sequencing the entire gene. For example, if a study identifies 2 different variants in *MYH7,* and then statistically tests the enrichment of both the variants in hypertrophic cardiomyopathy cases over unaffected controls, an aggregate variant analysis was conducted.
2. Case-control studies should be assigned points at the discretion of expert opinion based on the overall quality of each study. Assign each study a number of points between 0-6, then sum the points given to all studies, and fill in "S". NOTE: If the points given exceed the max score, use the Max score found in "T" for the summary matrix.
3. The quality of each case-control study should be evaluated using the following criteria in aggregate:
    a. **Variant Detection Methodology:** Cases and controls should ideally be analyzed using methods with equivalent analytical performance (e.g. equivalent genotype methods, sufficient and equivalent depth and quality of sequencing coverage).
    b. **Power:** The study should analyze a number of cases and controls given the prevalence of the disease, the allele frequency, and the expected effect size in question to provide appropriate statistical power to detect an association. (**NOTE:** The curator is NOT expected to perform power

calculations, but to record the information listed in this section for expert review.)

c. **Bias and Confounding factors:** The manner in which cases and controls were selected for participation and the degree of case-control matching may impact the outcome of the study. The following are some factors that should be considered:
    i. Are there systematic differences between individuals selected for study and individuals not selected for study (i.e. do the cases and controls differ in variables other than genotype)?
    ii. Are the cases and controls matched by demographic information (e.g., age, ethnicity, location of recruitment, etc.)? Are the cases and controls matched for genetic ancestry, if not did investigators account for genetic ancestry in the analysis?
    iii. Have the cases and controls been equivalently evaluated for presence or absence of a phenotype, and/or family history of disease?
d. **Statistical Significance**: The level of statistical significance should be weighed carefully.
    i. When an odds ratio (OR) is presented, its magnitude should be consistent with a monogenic disease etiology.
    ii. When p-values or 95% confidence intervals (CI) are presented for the OR, the strength of the statistical association can be weighed in the final points assigned.
    iii. Factors, such as multiple testing, that might impact that interpretation of uncorrected p-values and CIs should be considered when assigning points.

NOTE: Point totals should NOT exceed the max score. If the totals from "H-Q" exceed the max score, use the max score found in "U" for the genetic evidence portion of the summary matrix. Please prioritize curating genetic evidence over experimental evidence to reach a definitive score.

Figure 7: Case-control Genetic Evidence Examples

Detailed explanations for assigned points are provided below the table.

CASE-CONTROL DATA						
**Points**	**Power**	**Bias/ Confounding**	**Detection Method**	**Statistical Significance**	**Study Type**	**Points (0-6/ study)**
Author A 2015 (Max score)	Breast cancer cases: 100/12,000 Controls: 7/4,500	Matched by age, ethnicity, and location	Cases & controls genotyped for c.1439delA in gene *W*	OR: 5.4 [95% CI: 2.5-11.6; *P* < 0.0001]	Single Variant	6

Author B 2005 (Intermediate score)	HCM Cases: 13/200<br>Controls: 20/900	Matched by location, but not age or ethnicity	Cases & controls genotyped for p.Arg682Gln in gene *X*	Fisher's exact test<br>*P* = 0.004	Single Variant	4
Author C 2011<br>(Low score)	Ovarian cancer cases: 11/1,500<br>Controls: 3/2,000	Matched by ethnicity. Controls from population database (e.g. ExAC)	<u>Cases:</u> sequenced Gene *Y* and counted all cases with null variants.<br><u>Controls:</u> total individuals from population database with null variants in gene *Y*.	OR of all variants in aggregate: 4.9 (CI: 1.4-17.7; *P* =0.015)	Aggregate analysis	2
Author D 2009<br>(No case-control score)	Colorectal cancer cases: 11/1,500<br>Controls: 3/2,000	Matched by ethnicity. Controls from population database (e.g. ExAC)	<u>Cases:</u> sequenced gene *Z* and identified p.Lys342Ter in 11 cases.<br><u>Controls:</u> total individuals from population database with p.Lys342Ter in gene Z.	OR of p.Lys342: 4.9 (CI: 1.4-17.7; *P* =0.015)	Not applicable	0

Study receiving the max score (6 points): This single-variant analysis could receive the full 6 points based on the number of appropriately matched (i.e. no Bias or Confounding factors in study design) cases and controls analyzed (i.e. Power was sufficient given the prevalence of breast cancer as a disease) and the OR was highly statistically significant (P<0.0001) with a 95% CI that did not cross 1.0.

Study receiving intermediate score (4 points): This single-variant analysis could receive 4 points since the controls were not appropriately matched to the cases (i.e. by location alone and neither by ethnicity nor age) and the p-value is moderately significant.

Study receiving low score (2 points): This study is considered an aggregate analysis since the statistical test analyzed the variants in aggregate across all cases and controls. This study can be assigned 2 points because a population database was used rather than appropriately-matched controls (i.e. the study is not matched demographically) and the p-value is not very significant. A population database could be used as controls for 2 reasons:

a. Both the cases and controls were sequenced for the entire gene *Y*.
b. The total number of individuals with null variants (i.e. nonsense, canonical splice-site, and frameshift) was compared between cases and controls.

Study receiving no score (0 points): While this study is similar to the study receiving 2 points, the detection method differed between cases and controls (i.e. cases were sequenced, controls were genotyped). In the cases, gene *Z* was sequenced. However, only the controls with a specific variant were used for comparison to the cases.

Although this study cannot be counted as case-control data, it can be counted as case-level data.

EXPERIMENTAL EVIDENCE

Figure 8: Experimental Evidence Summary Matrix

EXPERIMENTAL EVIDENCE SUMMARY					
**Evidence Category**	**Evidence Type**	**Suggested Points/ Default**	**Range**	**Points Given**	**Max Score**
**Function**	Biochemical Function	A 0.5	0-2	L	W 2
	Protein Interaction	B 0.5	0-2	M	
	Expression	C 0.5	0-2	N	
**Functional Alteration**	Patient cells	D 1	0-2	O	X 2
	Non-patient cells	E 0.5	0-1	P	
**Models**	Non-human model organism	F 2	0-4	Q	Y 4
	Cell culture model	G 1	0-2	R	
**Rescue**	Rescue in human	H 2	0-4	S	
	Rescue in non-human model organism	I 2	0-4	T	
	Rescue in cell culture model	J 1	0-2	U	
	Rescue in patient cells	K 1	0-2	V	
**Total Allowable Points for Experimental Evidence**					**Z 6**

NOTE: Validated functional assays should be identified by expert panels or, if they are curator identified, confirmed by expert review.

Identify the experimental evidence type and assign points according to the following criteria.

1. Biochemical Function: Evidence showing the gene product performs a **biochemical function** shared with other known genes in the disease of interest, or consistent with the phenotype. NOTE: The biochemical function of both gene products must have been proven experimentally, and not just predicted. When awarding points in this evidence category, the other known gene(s) should have compelling evidence to support the gene-disease association. Consider increasing points based on the strength of the evidence and number of other proteins with the same function that are involved in the same disease. The suggested points/evidence can be found in column "A". Total up all of the experimental points and place them in the points given section found in "L".

2. Protein Interaction: Evidence showing the gene product **interacts** with **proteins previously implicated** in the disease of interest. Typical examples of this data include, but are not limited to: Physical interaction via Yeast-2-Hybrid (Y2H), co-immunoprecipitation (coIP), etc. NOTE: The interaction of the gene products must have been proven experimentally, and not just predicted. Proteins previously implicated in the disease of interest should have compelling evidence to support the gene-disease association. Note: Some studies provide evidence that a variant in the gene of interest disrupts the interaction of the gene product with another protein. In these cases, the positive control, showing interaction between the two wild type proteins, can be counted as evidence of protein interaction. Points can also be awarded to case-level (variant) evidence or functional alteration for the variant disrupting the interaction. The suggested points/evidence can be found in column "B". Total up all of the experimental points and place them in the points given section found in "M".
3. Expression: Summarize evidence showing the gene is expressed in **tissues relevant to the disease of interest** and/or is **altered in expression in patients** who have the disease. Typical examples of this data type are methods to detect a) RNA transcripts (RNAseq, microarrays, qPCR, qRT-PCR, Real-Time PCR) b) protein expression (western blot, Immunohistochemistry). Expert reviewers may specify appropriate uses of this category in the context of their particular disease domain. For example, groups may choose to award points based on the specificity of expression in relevant organs.
The suggested points per evidence can be found in column "C". Total up all of the experimental points and place them in the points given section found in "N". **NOTE:** If the sum of all biochemical function, protein interaction, and expression points exceeds the max score of 2, use the Max score found in "W" of the experimental evidence summary matrix.
4. Functional Alteration: Evidence showing the gene and/or gene product **function** is demonstrably altered in cultured patient or non-patient cells carrying candidate variants. For instance, does disrupting the gene in cells have a phenotype similar to that in human patients? Examples include experiments involving gene knock-down, overexpression, etc. Divide the evidence according to the following subtypes:
    a. Was the experiment conducted in **patient cells**? The suggested points/evidence can be found in column "D". Total up all of the experimental points and place them in the points given section found in "O".
    b. Was the experiment conducted in **non-patient cells**? The suggested points/evidence can be found in column "E". Total up all of the

experimental points and place them in the points given section found in "P".
NOTE: If the sum of all functional alteration points exceeds the max score of 2, use the Max score found in "X" of the experimental evidence summary matrix.

5. Model System: A **non-human model organism** or **cell culture model** with a disrupted copy of the gene shows a phenotype consistent with the human disease state. Note: Cell culture models should recapitulate the features of the diseased tissue e.g. engineered heart tissue, or cultured brain slices. These results should be summarized accordingly:
   c. Was the gene disruption in a **non-human model organism**? The suggested points/evidence can be found in column "F". Total up all of the experimental points and place them in the points given section found in "Q".
   d. Was the gene disrupted in a **cell culture model**? The suggested points/evidence can be found in column "G". Total up all of the experimental points and place them in the points given section found in "R"
6. Rescue: Summarize evidence showing the **phenotype in humans** (i.e. patients with the condition), **non-human model organisms, cell culture models,** or **patient cells** can be rescued by exogenous wild-type gene or gene product. These results should be recorded accordingly:
   a. Was the rescue in a **human?** For example, successful enzyme replacement therapy for a lysosomal storage disease. The suggested points/evidence can be found in column "H". Total up all of the experimental points and place them in the points given section found in "S".
   b. Was the rescue in a **non-human model organism**? The suggested points/evidence can be found in column "I".
   Note: While the default points and range of points are the same for human and non-human model organism, consider awarding more points if the rescue was in a human. Total up all of the experimental points and place them in the points given section found in "T".
   c. Was the rescue in a **cell culture model** (*i.e.* a cell culture model engineered to express the variant of interest). The suggested points/evidence can be found in column "J". Total up all of the experimental points and place them in the points given section found in "U".

d. Was the rescue in **patient cells**? The suggested points/evidence can be found in column "K". Total up all of the experimental points and place them in the points given section found in "V".
NOTE: If the sum of all models and rescue points exceeds the max score of 4, use the Max score found in "Y" of the experimental evidence summary matrix.

Total up the total number of experimental evidence points from Rows "W-Y" and enter them on Row "Z". **NOTE:** If the total experimental evidence points exceed the max score, use the Max score of 6 points for the summary matrix. **Please prioritize curating genetic evidence over experimental evidence to reach a definitive score.**

For specific examples of different pieces of experimental evidence, please see Appendix B.

Variant evidence vs experimental evidence
Not all evidence supports the role of the gene in the disease. Therefore, the curator must carefully consider whether to count functional evidence in the experimental evidence section or in the case-level data section. Only evidence that supports the role of the gene in the disease should be counted in the experimental evidence section. Experimental evidence that does not directly support the role of the gene in the disease but indicates that the variant is damaging to the gene function can, instead, be used to increase points in the case-level data section. Some very general examples are given below. Please note that these examples are a guide. Each piece of evidence should be carefully considered when deciding on which category to assign points. These decisions should be discussed with experts in the disease area, if needed.

Variant evidence, general examples:

- Immunolocalization showing that the gene product is mislocalized in cells from a patient or in cultured cells. This would be counted as case-level evidence UNLESS mislocalization/accumulation of an altered gene product is a known mechanism of disease, in which case this evidence could be counted as experimental evidence (functional alteration).
- Mini-gene splicing assay or RT-PCR showing that splicing is impacted by a splice-site variant.
- A variant in a gene encoding an enzyme is expressed in cultured cells and enzyme activity is deficient.
- A variant is shown to disrupt the normal interaction of the gene product of interest (protein A) with another protein (protein B). NOTE: If protein B is implicated in the same disease, the interaction can be counted in experimental data (Function: protein interaction), and the lack of interaction due to the variant can be counted as case-level data.

Experimental evidence, general examples:

- A signaling pathway is known to be involved in the disease mechanism. Expression of a missense variant in cells shows that the gene product can no longer function as part of this pathway.
- The variant is shown to be associated with a known hallmark of the disease e.g. abnormal deposition or mislocalization of a gene product, abnormal contractility of cells etc., either in patient cells or cultured cells expressing the variant.
- Study showing enzyme deficiency in tissues of many patients, leading to conclusion that deficiency of the enzyme causes the disease e.g. early studies showing enzyme deficiency in individuals with a metabolic disorder.
- Any model organism with a variant initially identified in a human with the disorder.

CONTRADICTORY EVIDENCE

NOTE: This designation is to be applied at the discretion of clinical domain experts after thorough review of available evidence. The curator will collect the contradictory evidence and the classification (Disputed/Refuted) is to be determined by the clinical domain experts. Below are a few examples of contradictory evidence. Note that this list is not all-inclusive and if the curator feels that a piece of evidence offers evidence that does not support the gene-disease relationship, this data should always be recorded (Summary and PMIDs) and pointed out for expert review.

1. **Case-control data is not significant:** As case-control studies evaluate variants in healthy vs affected individuals, if there is no statistically significant difference in the variants between these groups, this should be marked as potentially contradictory evidence for expert review. **See case-control examples above (p.22, Fig. 7) NOTE:** Evidence contradicting a single **variant** as causative for the disease does not necessarily rule out the gene:disease relationship.

2. **Minor allele frequency is too high for the disease:** Many diseases have published prevalence, which can often be found in the GeneReviews entry. If ALL minor alleles in a gene are present in a specific population or the general population (ExAC, ESP, 1000Genomes) at a frequency that is higher than what is estimated for the disease, this could suggest lack of gene-disease relationship and should be marked as potentially contradictory evidence for expert review. **For example,** Adams-Oliver syndrome is an autosomal dominant disease and has a prevalence of 0.44 in 100,000 (4.4e-6) live births. If a new gene were being curated for this disease and supposedly pathogenic variants were identified with an allele frequency in ExAC of 0.4882, this could be

potentially contradictory evidence. **NOTE:** Evidence contradicting a single **variant** as causative for the disease does not necessarily rule out the gene:disease relationship. Additionally, disease prevalence can vary in different populations, so read the GeneReviews entry thoroughly and keep demographic information in mind during this evaluation.

3. **The gene-disease relationship cannot be replicated:** One measure of a gene-disease relationship is its replication both over time and across multiple studies and disease cohorts. If a study could not identify any variants in the gene being curated in an affected population that was negative for other known causes of the disease, this could be considered potentially contradictory evidence and should be marked for expert review. **However,** when assigning this designation, a curator need consider disease prevalence. If a disease is rare, a small study may not identify any variants in the curated gene. **For example,** Perrault syndrome is characterized by hearing loss in males and ovarian dysfunction in females and only 100 cases have been reported. Thus, if a study with a small cohort does not identify any variants in a gene being curated for this syndrome, this may not necessarily be evidence against gene-disease association. In any case, if a curator suspects that any evidence supports a lack of gene-disease association, it should be marked for expert review.

4. **Non-segregations:** Non-segregations should be considered carefully, as age-dependent penetrance and phenotyping of relatives could have an impact on the number of apparent non-segregations within a family. Thus, the age of unaffected variant carriers should be of similar age to the affected variant carriers. If a curator suspects non-segregations, these should be noted for expert review.

5. **Non-supporting functional evidence:** The types of different experimental evidence are detailed in the **"Experimental Evidence" Section (p. 23)**. If any of this experimental evidence suggests that variants, although found in humans, do not affect function or that the function is not consistent with the established disease mechanism, this evidence should be marked as potentially contradictory evidence for expert review. **For example**, if a gene were being curated for a disease association and the mouse model did not have any phenotype, this could be potentially contradictory evidence.

SUMMARY AND FINAL MATRIX

A summary matrix was designed to generate a “provisional” clinical validity assessment using a point system consistent with the qualitative descriptions of each classification. This final gene curation matrix and instructions for filling it out can be found below.

Fill in the "Gene/Disease Pair" at the top of the matrix.

1. Enter the score calculated for Genetic Evidence Matrix (Fig. 3 p. 11) in row "A".
2. Enter the score calculated for Experimental Evidence Matrix (Fig. 8 p. 24) in row "B".
3. The sum of A and B is entered in row "C".
4. Refer to the publication date of the original publication of the gene-disease relationship and consider all other literature to complete row "D":
    a. YES if > 3 years have passed since the original publication AND there are >2 publications about the gene-disease relationship
    b. NO if >3 years have passed, BUT not >2 publications
    c. NO if < 3 years have passed
5. If there is valid contradictory evidence (see p. 28), compile this and briefly describe it (including the PubMed ID numbers) in row "E".
6. Choose the clinical validity classification associated with the value of the total points/replication over time (Row C, Row D) and complete row "F".
   NOTE: No matter the score, if there is contradictory evidence present, the curator classification must be listed as "Conflicting Evidence reported". The conflicting evidence will be weighed and reviewed by a domain expert.
7. When the gene-disease curation is reviewed by an expert, the expert will fill out the final classification in row "G".

Figure 9: Clinical Validity Summary Matrix

GENE/DISEASE PAIR:				
**Assertion criteria**	**Genetic Evidence (0-12 points)**	**Experimental Evidence (0-6 points)**	**Total Points (0-18)**	**Replication Over Time (Y/N)**
**Description**	Case-level, family segregation, or case-control data that support the gene-disease association	Gene-level experimental evidence that support the gene-disease association	Sum of Genetic & Experimental Evidence	> 2 pubs w/ convincing evidence over time (>3 yrs)
**Assigned Points**	A	B	C	D
**CALCULATED CLASSIFICATION**		**LIMITED**	**1-6**	
		**MODERATE**	**7-11**	
		**STRONG**	**12-18**	
		**DEFINITIVE**	**12-18 & Replicated Over Time**	
**Valid contradictory evidence (Y/N)***	**List PMIDs and describe evidence:** E			
**CURATOR CLASSIFICATION**		F		
**FINAL CLASSIFICATION**		G		

References:

1. **Strande, N.T., et al., *Evaluating the Clinical Validity of Gene-Disease Associations: An Evidence-Based Framework Developed by the Clinical Genome Resource*. Am J Hum Genet. 100(6): p. 895-906.**
2. **MacArthur, D.G., et al., *Guidelines for investigating causality of sequence variants in human disease*. Nature. 508(7497): p. 469-76.**
