# Supplementary material for: Evaluating the Clinical Validity of Hypertrophic Cardiomyopathy Genes
Source: Circ Genom Precis Med. 2019 Feb 19;12(2):e002460. doi: 10.1161/CIRCGEN.119.002460 (PMC6410971; doi:10.1161/CIRCGEN.119.002460)
Supplement: Supplementary file 3 [file hcg-12-e002460-s003.pdf]

Doi: 10.1161/CIRCGEN.119.002460

Evaluating the Clinical Validity of Hypertrophic Cardiomyopathy Genes

Running title: *Ingles et al.; HCM Gene Curation using the ClinGen Framework*

Jodie Ingles, GradDipGenCouns, PhD, MPH[1,2]; Jennifer Goldstein, PhD, CGC[3]; Courtney Thaxton, PhD[3]; Colleen Caleshu, MSc[4]; Edward W. Corty, MS3[3]; Stephanie B. Crowley, PhD[3]; Kristen Dougherty, MS[5]; Steven M. Harrison, PhD[6]; Jennifer McGlaughon, PhD[3] Laura V. Milko, PhD[3]; Ana Morales, MS, CGC[7]; Bryce A. Seifert, PhD[3]; Natasha Strande, PhD[3]; Kate Thomson, BSc, FRCPath[8]; J. Peter van Tintelen, MD, PhD[9]; Kathleen Wallace, BA[3]; Roddy Walsh, PhD[10,11]; Quinn Wells, MD, PharmD, MSc[12]; Nicola Whiffin, PhD[10,11]; Leora Witkowski, PhD[14]; Christopher Semsarian, MBBS, PhD, MPH[1,2]; James S. Ware MRCP PhD[10,11]; Ray E. Hershberger, MD[7,13]*; Birgit Funke, PhD, FACMG[14]*

[1]Agnes Ginges Centre for Molecular Cardiology at Centenary Institute & Faculty of Medicine and Health, University of Sydney; [2]Dept of Cardiology, Royal Prince Alfred Hospital, Sydney, Australia; [3]Dept of Genetics, UNC Chapel Hill, NC; [4]Stanford Center for Inherited Cardiovascular Disease, Stanford University, Stanford, CA; [5]Eastern Virginia Medical School, Norfolk,VA; [6]Laboratory for Molecular Medicine, Partners Healthcare, Harvard Medical School, Cambridge, MA; [7]Division of Human Genetics & Davis Heart and Lung Research Institute, [13]Division of Cardiovascular Medicine, The Ohio State University, Columbus, OH; [8]Oxford Medical Genetics Laboratory, Oxford, United Kingdom; [9]Dept of Clinical Genetics, Amsterdam University Medical Centers, University of Amsterdam, Cardiovascular Sciences, Amsterdam, Netherlands; [10]National Heart and Lung Institute & MRC London Institute of Medical Sciences, Imperial College London; [11]Cardiovascular Research Centre at Royal Brompton & Harefield Hospitals NHS Trust, London, United Kingdom; [12]Dept of Medicine, Vanderbilt University Medical Center, TN, [14]Department of Pathology, Harvard Medical School/Massachusetts General Hospital, Boston, MA
*contributed equally

American Heart Association

Correspondence:

Doctor Jodie Ingles

Agnes Ginges Centre for Molecular Cardiology

Centenary Institute, Locked Bag 6 Newtown NSW

2042 Sydney Australia

Tel: 02 9665 6100

E-mail: j.ingles@centenary.org.au

Twitter: @jodieingles27

Journal Subject Terms: Genetics; Cardiomyopathy

Abstract:

Background: Genetic testing for families with hypertrophic cardiomyopathy (HCM) provides a significant opportunity to improve care. Recent trends to increase gene panel sizes often mean variants in genes with questionable association are reported to patients. Classification of HCM genes and variants is critical, as misclassification can lead to genetic misdiagnosis. We show the validity of previously reported HCM genes using an established method for evaluating gene-disease associations.

Methods: A systematic approach was used to assess the validity of reported gene-disease associations, including associations with isolated HCM and syndromes including left ventricular hypertrophy (LVH). Genes were categorized as having definitive, strong, moderate, limited or no evidence of disease causation. We also reviewed current variant classifications for HCM in ClinVar, a publicly available variant resource.

Results: Fifty-seven genes were selected for curation based on their frequent inclusion in HCM testing and prior association reports. Of 33 HCM genes, only 8 (24%) were categorized as definitive (*MYBPC3, MYH7*, *TNNT2*, *TNNI3, TPM1*, *ACTC1*, *MYL2*, *MYL3);* 3 had moderate evidence (*CSRP3*, *TNNC1*, *JPH2;* 33%); and 22 (66%) had limited (n=16) or no evidence (n=6). There were 12 of 24 syndromic genes definitively associated with isolated LVH. Of 4191 HCM variants in ClinVar, 31% were in genes with limited or no evidence of disease association.

Conclusion: The majority of genes previously reported as causative of HCM and commonly included in diagnostic tests have limited or no evidence of disease association. Systematically curated HCM genes are essential to guide appropriate reporting of variants and ensure best possible outcomes for HCM families.

American Heart Association. Circulation: Genomic and Precision Medicine

Key words: hypertrophic cardiomyopathy; genetic testing; sarcomere; left ventricular hypertrophy; gene curation, misclassification

Introduction

Hypertrophic cardiomyopathy (HCM) is an inherited cardiomyopathy, characterized by left ventricular hypertrophy (LVH) in the absence of loading conditions such as hypertension.[1, 2] HCM affects approximately 1 in 500 in the general population[3, 4] with clinical features in patients ranging from asymptomatic to heart failure and sudden cardiac death. Since the first chromosomal location was mapped in 1989,[5] variants in numerous genes have been reported to cause HCM. Clinical diagnostic genetic testing for HCM has become increasingly part of mainstream clinical management of patients[6-8] with a key role in cascade testing of family members. Genotype positive relatives can be targeted for ongoing cardiac screening while genotype negative relatives can be released from life-long surveillance and worry.[8] While there is potential for HCM genetic testing to add significant value to family management, non-judicious use has potential for harm including variant misclassification and genetic misdiagnosis.[9]

American Heart
Circulation: Genomic and Precision Medicine

The implementation of next generation sequencing has led to a rapid expansion in the number of genes included in a typical diagnostic gene panel. Gene selection, in addition to increased stringency and expert classification of variants,[8-11] is a crucial but often overlooked first step. Identification of variants in genes with limited gene-disease association has the potential to add uncertainty and misinterpretation of variants as causative.[12-14] Here we examine the evidence supporting 57 genes included on diagnostic HCM gene panels using the NIH-funded Clinical Genome Resource (ClinGen) framework for evaluating gene-disease clinical validity.[15, 16] While other recent studies have investigated specific aspects of HCM gene association such as gene burden, we bring together all available evidence in a systematic way. We evaluate the quantity and quality of clinical genetic and experimental data using a scoring matrix and give a final overall summary classification. In addition, we use the public repository

ClinVar,[17] to cross-reference HCM variant classifications and to examine the impact of including insufficiently supported genes in clinical testing.

Methods

All data and materials have been made publicly available on the ClinGen website https://www.clinicalgenome.org/working-groups/clinical-domain/cardiovascular-clinical-domain-working-group/hypertrophic-cardiomyopathy-gene-ep/ and can be accessed at [URL of supplementary material]. No institutional review board approval was required. The full methods are available in the supplementary material.

Results

American Heart Association
Circulation: Genomic and Precision Medicine

Selection of the gene list

Of 328 gene panels identified in the NCBI Genetic Testing Registry, 24 were included (Supplementary Table 1; 20 panels identified as “HCM” panels and 4 “cardiomyopathy” panels). The mean (± standard deviation) number of genes per panel was 33 ± 25, including 8 ± 0.3 sarcomere genes, 4 ± 2 storage cardiomyopathy and RASopathy genes (*LAMP2*, *PRKAG2*, *TTR*, *GLA*, *PTPN11*, *RIT1*, *RAF1)* and 22 ± 23 “other” genes (range: 0-75). There were 162 unique genes represented across all panels (Supplementary Figure 2; Supplementary Table 2). Only 23 (14%) genes were present on >50% of the panels. All panels included key sarcomere genes (*MYBPC3*, *MYH7*, *TNNT2*, *TNNI3*, *TPM1*, *ACTC1*, *MYL2*, *MYL3*), except one that did not include *MYH7*. The final curation list included 57 genes (Supplementary Table 3 and 4); 33 were curated for HCM and 24 for syndromes or conditions including LVH (Supplementary Figure 3,

Supplementary Table 5). Twenty-six of the 57 genes had prior reported association with HCM in Online Mendelian Inheritance in Man (OMIM) (www.omim/org; Supplementary Table 6 and 7).

Classification of HCM genes

Of the 33 genes classified for HCM, 8 (24.2%) were classified as definitive, three (9.1%) as moderate, 16 (48.5%) as limited and 6 (18.2%) as no evidence (Figure 1). Those classified as definitive included well-known disease genes that have been included in diagnostic gene panels for over a decade (*MYBPC3, MYH7, TNNT2, TNNI3, TPM1, ACTC1, MYL3* and *MYL2*). All definitive genes primarily reached this classification due to genetic evidence reflecting numerous reports from the literature of causative variants in cases with HCM. Furthermore, all genes had some variants with strong segregation data and an aggregate variant excess in cases compared to controls (Table 1).[12] Moderate level gene classifications included *TNNC1, JPH2* and *CSRP3,* with evidence typically including either segregation evidence or reported *de novo* variants and some experimental evidence (Table 2). The majority (n=22, 66.7%) of genes had limited or no evidence of HCM association. Limited evidence genes typically included evidence from candidate gene studies, with observation of rare variants in cases, but without statistical evidence of an excess of rare variation in cases compared to background variation in controls, or segregation data, and minimal experimental evidence mostly from expression data. In some cases, limited evidence was available from animal models (*KLF10*, *MYOZ2*, *MYPN*).

Circulation: Genomic and Precision Medicine

Impact of including HCM candidate genes in diagnostic gene panels

The ACMG/AMP variant classification framework requires substantial evidence for a gene-disease association in order to assign a “pathogenic” or “likely pathogenic” classification to variants identified in any specific gene.[10] The inclusion of insufficiently supported genes in diagnostic testing practice increases the likelihood of inconclusive results being provided to

clinicians. Moreover, this effect can be pronounced when these genes have a high rate of population variation. To investigate this, we analyzed ClinVar variant entries for the HCM genes included in our curation effort. This resulted in 4191 assertions for variants in 50 genes (Supplementary Table 8). Of all assertions, 831 (20%) were classified as pathogenic, 584 (14%) were likely pathogenic and 2776 (66%) were VUS (Figure 2; Supplementary Table 9). There were 65 (5%) variants in genes with limited or no evidence classified as likely pathogenic or pathogenic, with most (n=38) being truncating or splice variants in *TTN*, which are known to occur at high frequency (~1%) in the general population. In total, 1252 VUS assertions were in HCM genes adjudicated as limited or no evidence and accounted for 30% of all assertions in ClinVar meeting analysis criteria for this study.

American Heart Association. Circulation: Genomic and Precision Medicine

Classification of syndromic genes

24 genes were curated for syndromes involving LVH, with the specific syndrome the gene was curated for is shown in Table 3. Two genes (*ACTN2*, *PLN*) best fit an intrinsic (primary) cardiomyopathy phenotype given there were no extracardiac features reported. *PLN* reached a definitive classification, with the phenotype spectrum including HCM, arrhythmogenic right ventricular cardiomyopathy and dilated cardiomyopathy (DCM).[18] *ACTN2* reached a moderate classification, with the reported phenotypes including HCM, left ventricular non-compaction (LVNC), atrial arrhythmias and idiopathic ventricular fibrillation.[19, 20] DCM cases were carefully reviewed to exclude end-stage HCM.

Twelve genes were curated for syndromes that may present with isolated, or seemingly isolated, LVH. Of those, 11 were classified as having a definitive association with their respective syndromes. *CACNA1C* is associated with Timothy syndrome. Infants may present with severe bi-ventricular hypertrophy, and two variants at a single amino acid position

(p.Arg518Cys and p.Arg518His) have been reported to occur in families with LVH, prolonged QT interval and sudden cardiac death.[21] Variants in *DES* cause a desminopathy, often occur *de novo* and are associated with a range of features including progressive skeletal muscle weakness, cardiomyopathy, including LVH in some cases, and cardiac conduction disease.[22] *FHL1* has been shown to cause Emery-Dreifuss muscular dystrophy, which can include LVH, even in the absence of significant muscle weakness.[23] *FLNC* causes a myofibrillar myopathy, though families with isolated LVH are known.[24, 25] Four genes, all associated with metabolic storage phenotypes that can mimic HCM (*GLA*: Fabry disease, *LAMP2*: Danon disease, *PRKAG2*: PRKAG2-cardiomyopathy, *TTR*: transthyretin amyloidosis), were classified as definitive. *PTPN11*, *RAF1* and *RIT1* are associated with Noonan syndrome and were classified as definitive. Autosomal recessive loss-of-function variants in *ALPK3* cause a severe infant-onset cardiomyopathy and were classified as strong.[26]

Ten genes were curated for syndromes that may include LVH only in combination with overt extra cardiac phenotypic features, therefore making variants in these genes unlikely to be reasonably mistaken for typical HCM (Table 3).

American Heart Association
Circulation: Genomic and Precision Medicine

Discussion

We report a systematic classification of genes commonly included in HCM genetic testing or previously reported as “HCM genes” in the public domain, using the ClinGen gene curation clinical validity framework.[15] Two-thirds of the curated genes had limited or no evidence of HCM association. We observed little consistency amongst currently offered diagnostic gene panels, likely influenced by the lack of widely accepted systematic curation efforts and absence of clear guidelines regarding the design of clinically valid gene panels. Reporting a VUS may

cause confusion, especially to clinicians less sanguine in understanding their marginal utility for clinical care. As genetic testing enters mainstream medical practice, discontinuity between evidence in support of specific gene-disease associations and the interpretation and use of variant data can be minimized with robust systematic gene classification efforts.

Our findings have direct clinical implications for the genetic evaluation and care of HCM families. We show that in ClinVar, a large publicly accessible database, nearly 30% of assertions made for HCM in our curated gene list were VUS in genes with limited or no evidence of HCM association. This illustrates VUS inflation due to reporting of variants in these genes. Genetic test results are probabilistic and some degree of uncertainty is inherent, though efforts should be made to minimize this where possible in the clinical setting, focusing on results that are meaningful to the clinician, patient and family. Given the uncertainty we face in understanding the significance of rare variation in numerous genes, the reporting of VUS in genes with tenuous HCM association is at best unnecessary and time consuming, and at worst has potential to inflict harm to families who may be over-investigated or inappropriately treated,[9, 27] especially in the absence of specialised disease-specific expertise. As predictive testing becomes mainstream, it will be increasingly important to consider the evidence supporting a disease association to avoid misclassification of uncertain variants that can lead to unnecessary medical action.

American Heart Association
Circulation: Genomic and Precision Medicine

Clearly defined boundaries are needed for when candidate genes should be utilized for clinical testing. The current plethora of genes purported to be associated with HCM is the result of dedicated efforts to better elucidate its genetic architecture. A decade ago, the promise of identifying new genes to explain gene-negative HCM cases was the driver of numerous candidate gene studies. At that time a missense variant in a conserved region, absent in a small set of control alleles, and with some evidence of segregation, suggested a new HCM gene

association. Such an approach is now widely recognized as insufficient[10, 28] however, published disease associations have led to inclusion of such genes on clinical test panels.

Diagnostic gene panels should include genes considered to have definitive or strong evidence of disease association to minimize the risk of inconclusive findings. Moderately associated genes should be considered more carefully, though variants may be considered causative if there is very clear supportive evidence of a functional or damaging effect for the variant. Inclusion of genes with limited or no evidence for a disease association can be useful when the availability of large pedigrees allows clarification of uncertain variants identified in the proband, or when multiple family member exome or genome testing is used for clinically challenging cases. However, these applications currently straddle the boundary between diagnostic and research testing and their benefit needs to be carefully weighed against potential negative impact to the patient.

American Heart Association

Circulation: Genomic and Precision Medicine

Given isolated LVH may be confused with a diagnosis of HCM, inclusion of genes with moderate level and above for syndromes leading to isolated LVH is warranted. Where causative variants in syndrome genes are identified, concordance with the extra-cardiac phenotype features is important. Defining a precise cause of LVH, by identifying a genetic cause implicating an HCM mimic, has direct clinical advantages in many cases. For example, more targeted therapies such as enzyme replacement therapy in Fabry disease, or in guiding prognosis in young males with Danon disease. Appropriate recognition and implementation of such information by genetic testing groups and clinical genetics professionals is needed, and we suggest this process as a basis for laboratories to aid in designing gene panels that match their intended use. Increasingly, panels are configured to evaluate several disorders with clinical and genetic overlap, however this approach is likely to result in greater identification of VUS.

Study limitations include genes potentially not curated in this iteration. However, our systematic approach to selecting genes ensured that we included most with a published association with HCM and those commonly included on panels. The ClinGen gene curation framework assumes a Mendelian mode of inheritance; more complex models, should they be relevant, exceeded this analysis paradigm. Gene classifications provided were based on available evidence at the time of curation; ongoing and updated reassessments of gene-disease associations are essential. Importantly, our framework relies on variant curation, and while the difficulties in determining variant pathogenicity have been well documented,[12, 13, 29, 30] we relied on variant curation best practices as foundational to this effort. The materials and framework to perform gene curations are publicly available. Summaries for the gene-disease evidence assessments are available online (https://search.clinicalgenome.org/kb/gene-validity) and in Supplementary Document 2.

American Heart Association. Circulation: Genomic and Precision Medicine

Conclusion

HCM genetic testing has entered mainstream medical care. HCM genetic testing has important benefits for asymptomatic at-risk relatives and in the future may play a role in prognostic and therapeutic stratification of the proband. Our findings highlight that most reported "HCM genes" are spurious, including many genes routinely included in current diagnostic panels, with profound implications for the risk of genetic misdiagnosis in HCM families. Robust international gene curation efforts, as described here, bring together many types of evidence and are essential to yield the greatest value from HCM genetic testing.

Acknowledgments: With many thanks to Brandi Kattman, MS, CGC, Staff Scientist / Genetic Counselor, NIH Genetic Testing Registry for sharing data on genes present on HCM NGS panels. We would also like to thank various groups within ClinGen including the RASopathy

Expert Panel and Hearing Loss Working Group for input on genes of interest; the Lumping and Splitting Working Group for guidance on decisions regarding disease entities to curate; and the Gene Curation Working Group (co-chairs Jonathan Berg, MD, PhD, and Christa Martin, PhD, FACMG) for helpful discussions and guidance on the gene-disease clinical validity framework.

Sources of Funding: J Ingles is the recipient of a National Heart Foundation of Australia Future Leader Fellowship (#100833). JP van Tintelen acknowledges the support from the Netherlands Cardiovascular Research Initiative, an initiative with support of the Dutch Heart Foundation (CVON2014-40 DOSIS). K Thomson is funded by a National Institute for Health Research (NIHR) and Health Education England (HEE) Healthcare Science Doctoral Research Fellowship (NIHR-HCS-D13-04-006). C Semsarian is the recipient of a National Health and Medical Research Council (NHMRC) Practitioner Fellowship (#1059156). R Hershberger and A Morales were supported by ClinGen subcontracts (NHGRI HG007437; NHGRI 1U41HG009650). We are very grateful to the National Human Genome Research Institute (NHGRI) for funding this work (grant #U01HG007437-04).

American Heart Association
Circulation: Genomic and Precision Medicine

Disclosures: None.

References:

1. Gersh BJ, et al. 2011 ACCF/AHA Guideline for the Diagnosis and Treatment of Hypertrophic Cardiomyopathy: a report of the American College of Cardiology Foundation/American Heart Association Task Force on Practice Guidelines. Developed in collaboration with the American Association for Thoracic Surgery, American Society of Echocardiography, American Society of Nuclear Cardiology, Heart Failure Society of America, Heart Rhythm Society, Society for Cardiovascular Angiography and Interventions, and Society of Thoracic Surgeons. *J Am Coll Cardiol*. 2011;58:e212-260.

2. Elliott PM, et al. 2014 ESC Guidelines on diagnosis and management of hypertrophic cardiomyopathy: the Task Force for the Diagnosis and Management of Hypertrophic Cardiomyopathy of the European Society of Cardiology (ESC). *Eur Heart J*. 2014;35:2733-2779.

3. Semsarian C, et al. New perspectives on the prevalence of hypertrophic cardiomyopathy. *J Am Coll Cardiol*. 2015;65:1249-1254.

4. Maron BJ, et al. Prevalence of hypertrophic cardiomyopathy in a general population of young adults. Echocardiographic analysis of 4111 subjects in the CARDIA Study. Coronary Artery Risk Development in (Young) Adults. *Circulation*. 1995;92:785-789.

5. Jarcho JA, et al. Mapping a gene for familial hypertrophic cardiomyopathy to chromosome 14q1. *N Engl J Med*. 1989;321:1372-1378.

6. Maron BJ, et al. Hypertrophic Cardiomyopathy: Present and Future, With Translation Into Contemporary Cardiovascular Medicine. *J Am Coll Cardiol*. 2014;64:83-99.

7. Cirino AL, et al. Role of Genetic Testing in Inherited Cardiovascular Disease: A Review. *JAMA Cardiol*. 2017;2:1153-1160.

8. Hershberger RE, et al. Genetic evaluation of cardiomyopathy: a clinical practice resource of the American College of Medical Genetics and Genomics (ACMG). *Genet Med*. 2018;20:899-909.

9. Manrai AK, et al. Genetic Misdiagnoses and the Potential for Health Disparities. *N Engl J Med*. 2016;375:655-665.

10. Richards S, et al. Standards and guidelines for the interpretation of sequence variants: a joint consensus recommendation of the American College of Medical Genetics and Genomics and the Association for Molecular Pathology. *Genet Med*. 2015;17:405-424.

11. Kelly M, et al. Adaptation and Validation of the ACMG/AMP variant classification framework for MYH7-associated inherited cardiomyopathies: Recommendations by ClinGen's Inherited Cardiomyopathy Expert Panel. *Genet Med*. 2018;20:351-359.

12. Walsh R, et al. Reassessment of Mendelian gene pathogenicity using 7,855 cardiomyopathy cases and 60,706 reference samples. *Genet Med*. 2017;19:192-203.

13. Das KJ, et al. Determining pathogenicity of genetic variants in hypertrophic cardiomyopathy: importance of periodic reassessment. *Genet Med*. 2014;16:286-293.

14. Furqan A, et al. Care in Specialized Centers and Data Sharing Increase Agreement in Hypertrophic Cardiomyopathy Genetic Test Interpretation. *Circ Cardiovasc Genet*. 2017;10. pii: e001700. doi: 10.1161/CIRCGENETICS.116.001700.

15. Strande NT, et al. Evaluating the Clinical Validity of Gene-Disease Associations: An Evidence-Based Framework Developed by the Clinical Genome Resource. *Am J Hum Genet*. 2017;100:895-906.

16. Rehm HL, et al. ClinGen--the Clinical Genome Resource. *N Engl J Med*. 2015;372:2235-2242.

American Heart Association
Circulation: Genomic and Precision Medicine

17. Landrum MJ, et al. ClinVar: public archive of interpretations of clinically relevant variants. *Nucleic Acids Res*. 2016;44:D862-868.

18. van der Zwaag PA, et al. Phospholamban R14del mutation in patients diagnosed with dilated cardiomyopathy or arrhythmogenic right ventricular cardiomyopathy: evidence supporting the concept of arrhythmogenic cardiomyopathy. *Eur J Heart Fail*. 2012;14:1199-1207.

19. Chiu C, et al. Mutations in alpha-actinin-2 cause hypertrophic cardiomyopathy: a genome-wide analysis. *J Am Coll Cardiol*. 2010;55:1127-1135.

20. Girolami F, et al. Novel alpha-actinin 2 variant associated with familial hypertrophic cardiomyopathy and juvenile atrial arrhythmias: a massively parallel sequencing study. *Circ Cardiovasc Genet*. 2014;7:741-750.

21. Boczek NJ, et al. Identification and Functional Characterization of a Novel CACNA1C-Mediated Cardiac Disorder Characterized by Prolonged QT Intervals With Hypertrophic Cardiomyopathy, Congenital Heart Defects, and Sudden Cardiac Death. *Circ Arrhythm Electrophysiol*. 2015;8:1122-1132.

22. van Spaendonck-Zwarts KY, et al. Desmin-related myopathy. *Clin Genet*. 2011;80:354-366.

23. Knoblauch H, et al. Contractures and hypertrophic cardiomyopathy in a novel FHL1 mutation. *Ann Neurol*. 2010;67:136-140.

24. Gomez J, et al. Screening of the Filamin C Gene in a Large Cohort of Hypertrophic Cardiomyopathy Patients. *Circ Cardiovasc Genet*. 2017;10. pii: e001584. doi: 10.1161/CIRCGENETICS.116.001584.

25. Valdes-Mas R, et al. Mutations in filamin C cause a new form of familial hypertrophic cardiomyopathy. *Nat Commun*. 2014;5:5326.

26. Almomani R, et al. Biallelic Truncating Mutations in ALPK3 Cause Severe Pediatric Cardiomyopathy. *J Am Coll Cardiol*. 2016;67:515-525.

27. Ackerman JP, et al. The Promise and Peril of Precision Medicine: Phenotyping Still Matters Most. *Mayo Clin Proc*. 2016. pii: S0025-6196(16)30463-3. doi: 10.1016/j.mayocp.2016.08.008. [Epub ahead of print]

28. MacArthur DG, et al. Guidelines for investigating causality of sequence variants in human disease. *Nature*. 2014;508:469-476.

29. Whiffin N, et al. Using high-resolution variant frequencies to empower clinical genome interpretation. *Genet Med*. 2017;19:1151-1158.

30. Whiffin N, et al. CardioClassifier: disease- and gene-specific computational decision support for clinical genome interpretation. *Genet Med*. 2018;20:1246-1254.

American Heart Association
Circulation: Genomic and Precision Medicine

Table 1: Definitive classifications for HCM-gene associations

Gene	Variant evidence	Variant excess in cases	Segregation in families	Total Genetic Evidence (0-12)	Function	Functional Alteration	Models and Rescue	Total Experimental Evidence (0-6)	Replication over time	Total points	Classification
MYBPC3	7	2	1.5	11	2	0	4	6	Yes	17	Definitive
MYH7	7.5	2	3	12	1	2	2	5	Yes	17	Definitive
TNNT2	7	2	3	12	1.5	2	2.5	6	Yes	18	Definitive
TNNI3	9	2	1.5	12	2	0	4	6	Yes	18	Definitive
TPM1	8	2	3	12	0.5	2.5	3	6	Yes	18	Definitive
ACTC1	11	2	1.5	12	2	0.5	3	5.5	Yes	17.5	Definitive
MYL3	7	2	2.5	11.5	1.5	0	1	2.5	Yes	14	Definitive
MYL2	9	2	0.5	11.5	1.5	0	3	4.5	Yes	18	Definitive

American Heart Association.
Circulation: Genomic and Precision Medicine

Table 2: Genetic, experimental and overall classifications for genes curated for HCM

	Genetic Evidence	Experimental Evidence	Score	Classification	Presence on GTR panels, n (%)	ClinVar LP/P HCM submissions, n (%)	Inheritance pattern	Variant type*
MYL2	11.5	4.5	18	Definitive	24 (100)	33 (2.3)	AD	Missense
TNNT2	12	6	18	Definitive	24 (100)	71 (5.0)	AD	Missense, Splice
ACTC1	12	5.5	17.5	Definitive	22 (92)	11 (0.8)	AD	Missense
MYBPC3	11	6	17	Definitive	24 (100)	628 (44.4)	AD	Missense, LOF
MYH7	12	5	17	Definitive	23 (96)	393 (27.8)	AD	Missense
TPM1	11	6	17	Definitive	24 (100)	26 (1.8)	AD	Missense
TNNI3	10.5	6	16.5	Definitive	24 (100)	64 (4.5)	AD	Missense
MYL3	11.5	2.5	14	Definitive	24 (100)	16 (1.1)	AD	Missense, Splice
CSRP3	8.4	2.5	10.9	Moderate	16 (67)	8 (0.6)	AD	Missense, LOF
TNNC1	4	5	9	Moderate	22 (92)	8 (0.6)	AD	Missense, LOF
JPH2	4.2	4.5	8.7	Moderate	12 (50)	4 (0.3)	AD	Missense
TTN	1.5	4.5	6	Limited	10 (42)	42 (3.0)	AD	Missense
KLF10	1.25	4.5	5.75	Limited	1 (4)	0 (0)	AD	Missense
MYPN	0.9	2.5	3.4	Limited	9 (38)	2 (0.1)	AD	Missense, LOF
ANKRD1	1.1	2	3.1	Limited	10 (42)	1 (0.1)	AD	Missense
MYLK2	0.1	3	3.1	Limited	10 (42)	2 (0.1)	AD	Missense
MYOZ2	1.5	1.5	3	Limited	15 (63)	2 (0.1)	AD	Missense
NEXN	0.5	1.5	2	Limited	15 (63)	2 (0.1)	AD	Missense
VCL	0.5	1	1.5	Limited	12 (50)	3 (0.2)	AD	Missense
TRIM63	0.4	1	1.4	Limited	2 (8)	0 (0)	AD	Missense, LOF
RYR2	0.9	0.5	1.4	Limited	5 (21)	0 (0)	AD	Missense, LOF
MYH6	0.3	1	1.3	Limited	15 (63)	4 (0.3)	AD	Missense
OBSCN	0.3	1	1.3	Limited	2 (8)	0 (0)	AD	Missense, LOF
PDLIM3	0.2	1	1.2	Limited	4 (17)	0(0)	AD	Missense, LOF
TCAP	0.2	1	1.2	Limited	12 (50)	3 (0.2)	AD	Missense
MYOM1	0.5	0.5	1	Limited	2 (8)	1 (0.1)	AD	Missense
CALR3	0.2	0	0.2	Limited	7 (29)	1 (0.1)	AD	Missense
ACTA1	0	0	0	No evidence	1 (4)	0 (0)	-	-
CASQ2	0	0	0	No evidence	4 (17)	0 (0)	-	-
CACNB2	0	0	0	No evidence	2 (8)	0 (0)	-	-
DSP	0	0	0	No evidence	5 (12)	0 (0)	-	-
KCNQ1	0	0	0	No evidence	2 (8)	2 (0.1)	-	-
TMPO	0	0	0	No evidence	3 (13)	0 (0)	-	-

*Type of variant for those counted in genetic evidence. Other variant types may have been reported but not included in score if maximum number already reached. Loss of function (LOF) includes insertions and deletions leading to a frameshift, canonical splice and nonsense variants. Abbreviations: GTR, genetic testing registry; LP/P, likely pathogenic and pathogenic.

Circulation: Genomic and Precision Medicine

Table 3: Genetic, experimental and overall classifications for genes curated for syndromes including LVH

	Genetic Evidence	Experimental Evidence	Score	Classification	Phenotype Spectrum Curated	Isolated* LVH?	Presence on GTR HCM panels, n (%)
Intrinsic cardiomyopathy genes							
ACTN2	3.6	4.5	8.1	Moderate	LVH, LVNC, DCM, idiopathic VF	Yes	14 (58.3)
PLN	8.75	6	14.75	Definitive	HCM, DCM, ARVC	Yes	15 (62.5)
Syndromic genes, where isolated LVH may be seen							
ALPK3	9	3.5	12.5	Strong	Infant-onset HCM/DCM	Yes	1 (4)
CACNA1C	10.85	6	16.85	Definitive	Timothy syndrome	Yes	2 (8.3)
DES	12	6	18	Definitive	Desminopathy	Yes	8 (33.3)
FHL1	12	6	18	Definitive	Emery-Dreifuss MD	Yes	5 (20.8)
FLNC	7.5	6	13.5	Definitive	Myofibrillar myopathy	Yes	2 (8.3)
GLA	12	5	17	Definitive	Fabry disease	Yes	19 (79.2)
LAMP2	12	3.5	15.5	Definitive	Danon disease	Yes	20 (83.3)
PRKAG2	12	6	18	Definitive	*PRKAG2* cardiomyopathy	Yes	20 (83.3)
PTPN11	12	6	18	Definitive	Noonan syndrome	Yes	7 (29.2)
RAF1	12	6	18	Definitive	Noonan syndrome	Yes	7 (29.2)
RIT1	12	6	18	Definitive	Noonan syndrome	Yes	2 (8.3)
TTR	12	5.5	17.5	Definitive	Transthyretin amyloidosis	Yes	17 (70.8)
Syndromic genes, where LVH is seen only with overt syndromic features							
ABCC9	12	1	13	Definitive	Cantu syndrome	No	4 (16.7)
BAG3	7	5	12	Definitive	Myofibrillar myopathy	No	6 (25.0)
CAV3	8.5	6	14.5	Definitive	Caveolinopathy	No	15 (62.5)
COX15	8	5	13	Strong	Leigh syndrome	No	4 (16.7)
CRYAB	7	6	13	Definitive	Alpha-crystallinpathy	No	5 (20.8)
FXN	10	6	16	Definitive	Friedreich ataxia	No	3 (12.5)
GAA	12	6	18	Definitive	Pompe disease	No	4 (16.7)
LDB3	3.3	4.5	7.8	Moderate	Myofibrillar myopathy	No	9 (37.5)
MYO6	12	3	15	Definitive	Bilateral hearing loss, LVH †	No	0 (0)
SLC25A4	7.5	6	13.5	Definitive	Mitochondrial disease	No	3 (12.5)

*Includes seemingly isolated left ventricular hypertrophy; †One family reported to have 4/10 affected relatives also having LVH.
Abbreviations: LVH, left ventricular hypertrophy; LVNC, left ventricular noncompaction; ARVC, arrhythmogenic right ventricular cardiomyopathy; GTR, genetic testing registry; MD, muscular dystrophy; VF, ventricular fibrillation.

Circulation: Genomic and Precision Medicine

Doi: 10.1161/CIRCGEN.119.002460

Figure Legends:

Figure 1: HCM gene-disease classifications showing genetic, experimental and overall scores and classification

Figure 2: Number of ClinVar assertions for an HCM phenotype, grouped by gene classifications. Abbreviations: LVH, left ventricular hypertrophy.

American Heart Association

Circulation: Genomic and Precision Medicine

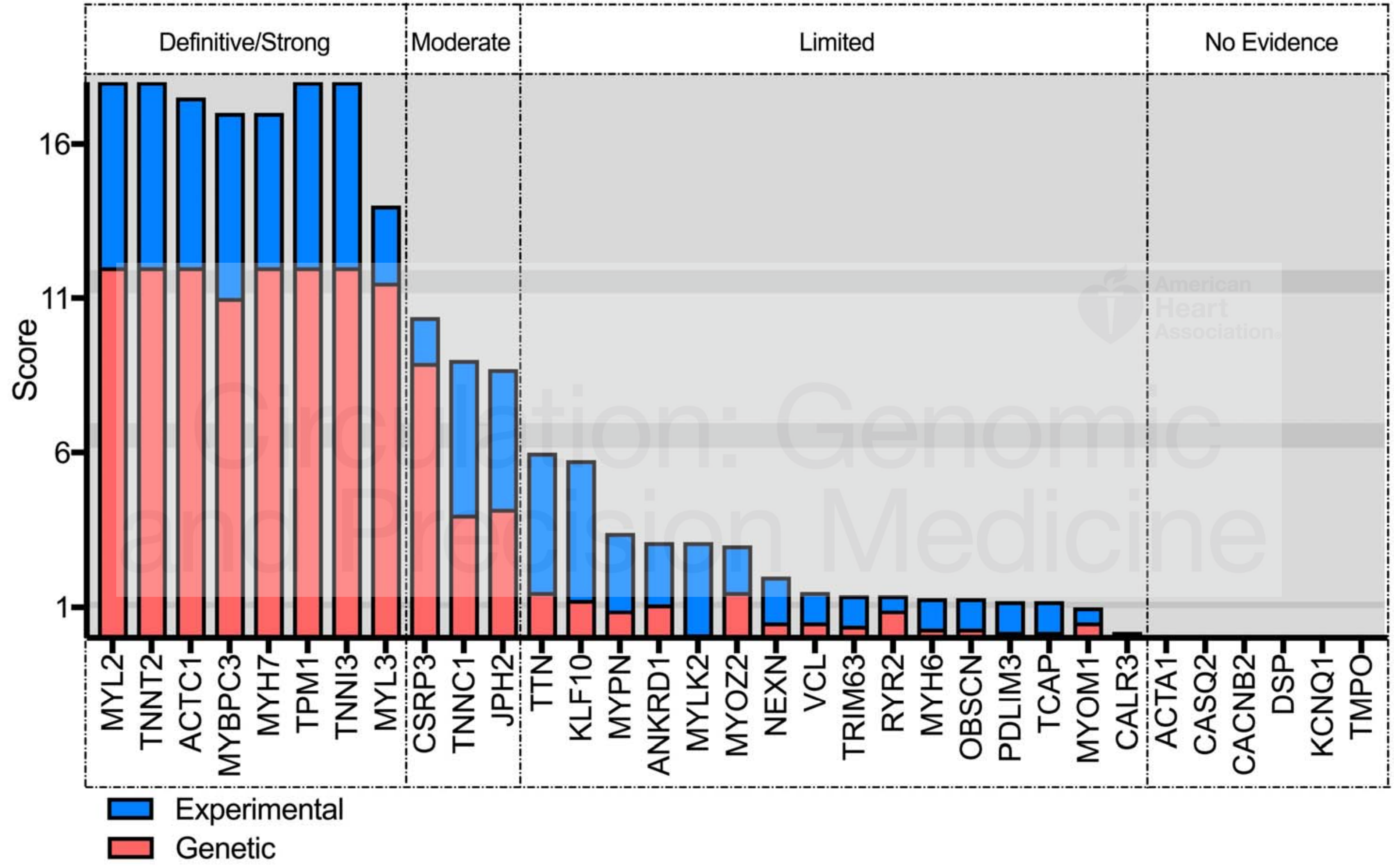

Circulation: Genomic and Precision Medicine
American Heart Association

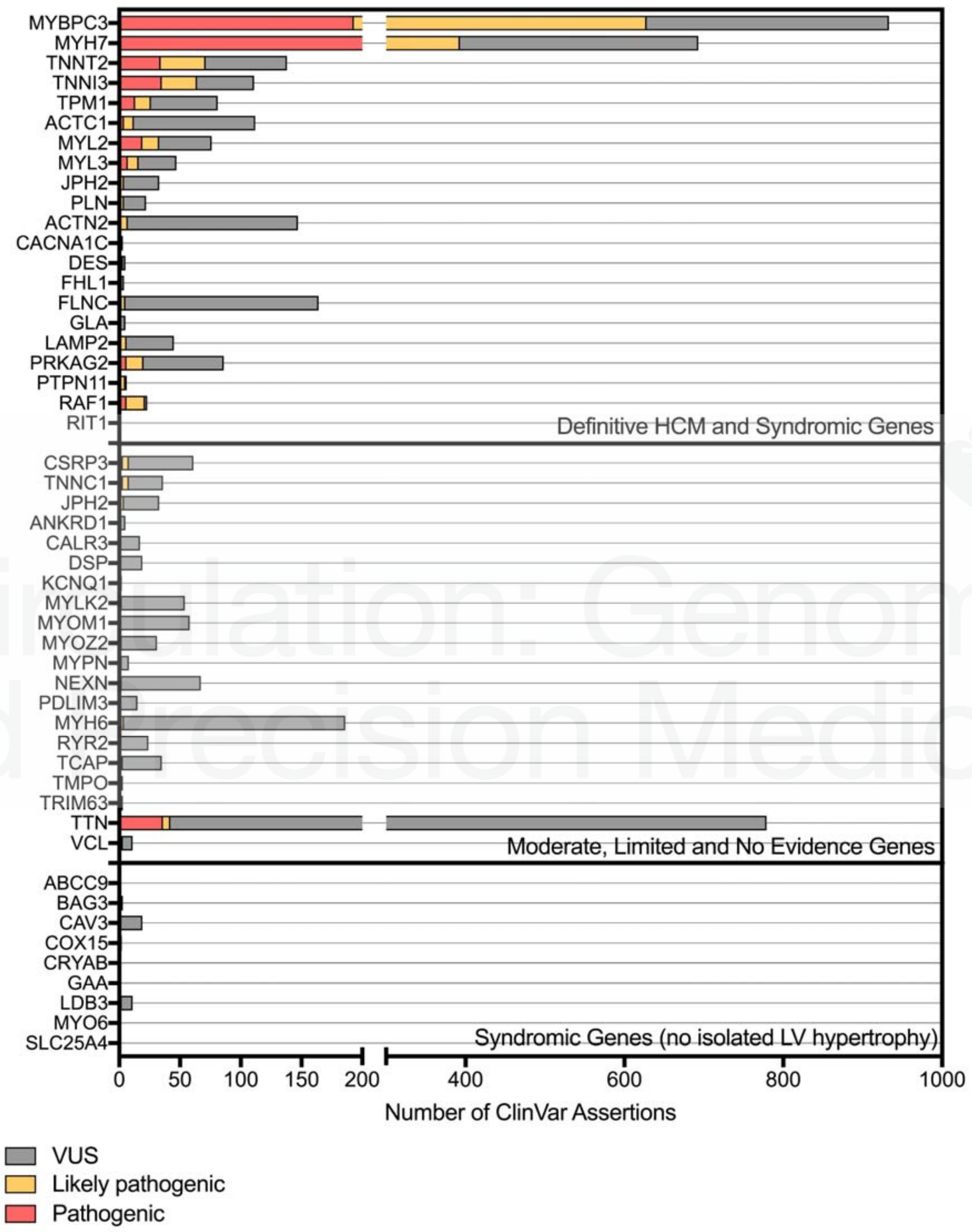

Circulation: Genomic and Precision Medicine
American Heart Association
